# Process Design for Small-Scale Production of PEGDA-NVP Hydrogel Nanoparticle Emulsions for Therapeutic Applications

**DOI:** 10.1021/acsomega.6c04988

**Published:** 2026-08-12

**Authors:** Ruth Negru, Fernando T. P. Borges, Georgia Papavasiliou, Fouad Teymour, Marcella K. Vaicik

**Affiliations:** † Department of Biomedical Engineering, 2455 Illinois Institute of Technology, Chicago, Illinois 60616, United States; ‡ Department of Chemical and Biological Engineering, Illinois Institute of Technology, Chicago, Illinois 60616, United States

## Abstract

Hydrogel nanoparticle emulsions (HNEs) are promising platforms for intracellular therapeutic delivery, yet their synthesis at bench scale (267 mL) requires large quantities of material, which limits iterative optimization with high-value cargos. Here, we established a small-scale inverse phase (water-in-oil, W/O) miniemulsion polymerization (IPMP) process (15 mL) that reduced material requirements by >90% while maintaining key process design criteria. Using poly­(ethylene glycol) diacrylate (PEGDA) and *n*-vinyl-2-pyrrolidinone (NVP), we demonstrate that the small-scale process maintains key measured formulation attributes, including nanoparticle tracking analysis (NTA)-derived particle diameter distributions, swelling ratios, swelling-derived mesh dimension estimations, and colloidal stability for at least three years. Process scale-down identified optimized parameters for homogenization, sonication, nitrogen purging, and reaction time and yielded nanoparticles with reproducible NTA-derived size metrics, concentration, and swelling-derived mesh dimension estimations. PEGDA-2000-based HNEs were cytocompatible according to International Organization for Standardization (ISO) 10993-5 exceeding the cytotoxic threshold (≥70% viability) under *en masse* administration. Fluorescently labeled HNE nanoparticles exhibited rapid internalization in adipocytes, which are typically resistant to transfection. With administration of HNEs loaded with green fluorescent protein (GFP) cargo, cells showed delayed appearance of GFP-associated fluorescence on day 8, consistent with time-dependent cargo release. Collectively, these findings establish a small-volume HNE synthesis platform that reduces material requirements while preserving evaluated formulation attributes across scales and demonstrating intracellular cargo delivery.

## Graphical abstract

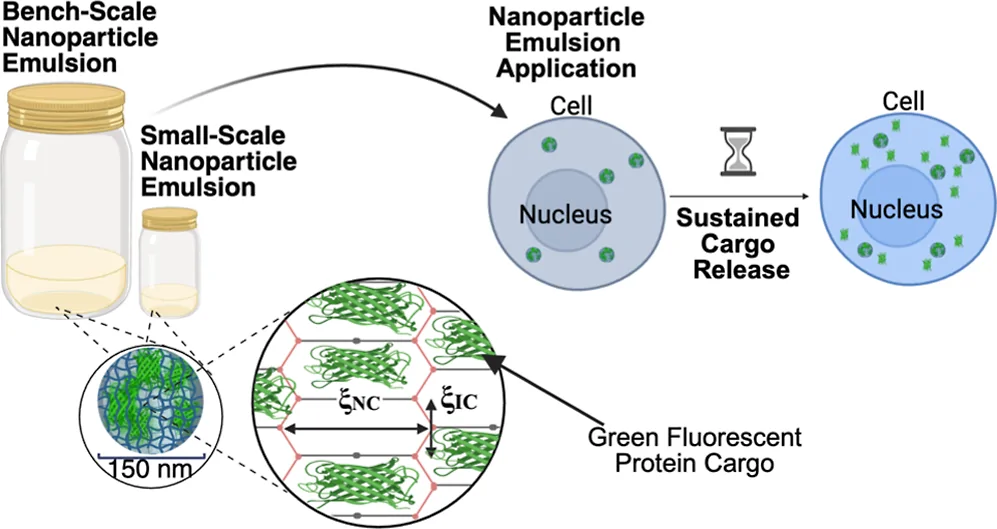

## 1 Introduction

Polymeric nanoparticles are widely used for therapeutic delivery because cross-linked network architectures provide tunable cargo encapsulation and diffusion-controlled release.^1–4^ In hydrogel nanoparticles (nanogels), diffusion-mediated release kinetics are governed by mesh architecture, swelling behavior, and cross-link density, establishing a direct structure–property relationship between network design and therapeutic performance.^5,6^ The poly­(ethylene glycol) diacrylate (PEGDA) and n-vinyl-2-pyrrolidinone (NVP) (PEGDA/NVP) hydrogel nanoparticles studied here conform to the Medical Subject Headings (MeSH) definition of nanogels: spherical, 20–250 nm cross-linked hydrophilic polymer particles designed for drug delivery.

Inverse phase miniemulsion polymerization (IPMP), historically developed for latex production, is now adapted for biomedical applications, where its ability to encapsulate hydrophilic cargo within nanoscale carriers is particularly valuable.^7^ We previously developed a bench-scale (267 mL) IPMP process that produces PEGDA/NVP hydrogel nanoparticles for hydrophilic therapeutic delivery.^4,8^ The underlying free-radical copolymerization mechanism and resulting network architecture have been characterized, including kinetic modeling of initiation, propagation, branching, and cross-link formation.^9^ PEGDA and NVP undergo free-radical copolymerization, in which NVP contributes to efficient chain growth and PEGDA provides multifunctional acrylate groups that generate branching and cross-link formation (Figure 1). Polymerization proceeds within aqueous nanodroplets dispersed in an oil phase. In prior bench-scale formulations, cyclohexane was used as the continuous oil phase, necessitating nanoparticle isolation prior to biological use due to cytotoxicity of cyclohexane.^4,10,11^ Although these hydrogel nanoparticles demonstrated therapeutic efficacy in multiple applications (sepsis prevention,^12^ biofilm inhibition,^13–15^ and promotion of neovascularization,^16^) postprocessing steps were associated with reduced particle recovery and burst cargo release, motivating continued process refinement.^17^

**Figure 1:**
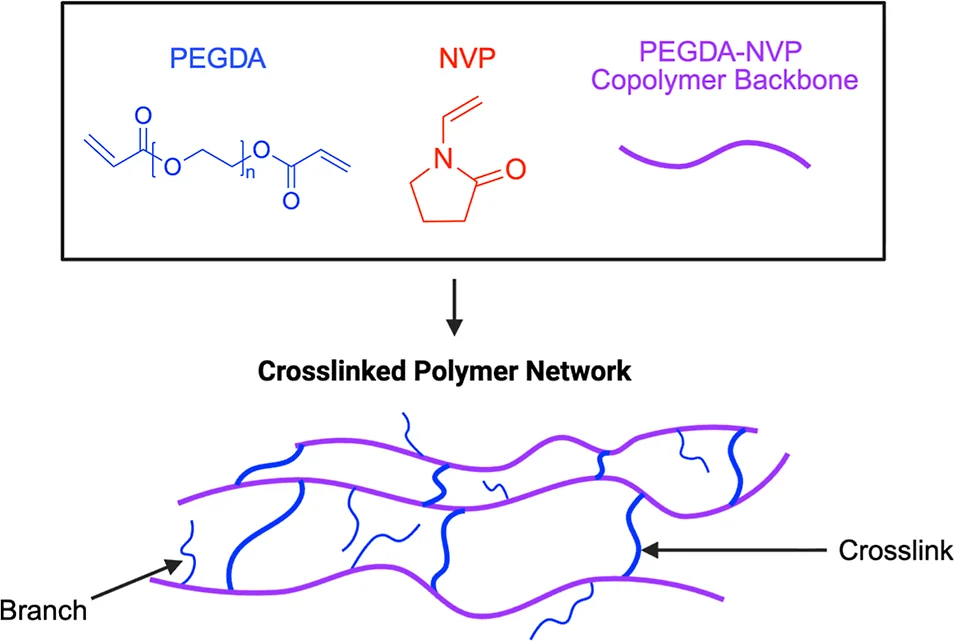
Schematic representation of PEGDA/NVP free-radical polymerization and network formation. Branch points and cross-links arise from PEGDA multifunctionality and are shown in blue. Created in BioRender. Vaicik, M. (2026) https://BioRender.com/a6_mga0j.

IPMP functions as a population of nanoconfined aqueous reactors dispersed within a continuous oil phase, where polymerization proceeds independently within spatially isolated droplets.^3,18^ Consequently, the resulting nanoparticle diameter, cross-link density, and network architecture are governed by droplet-scale transport and free-radical kinetics rather than total batch volume. Droplet size distribution is dictated by the balance between interfacial tension and applied shear or acoustic energy density,^19,20^ while cross-link formation is determined by intradroplet monomer composition and temperature–time exposure relative to initiator decomposition kinetics. Thus, the nanoscale properties of hydrogel nanoparticles are primarily influenced by droplet-confined polymerization conditions rather than total reactor volume under the conditions studied. Engineering nanoconfined polymerization for materials-efficient nanoscale optimization therefore requires maintaining these governing transport and kinetic conditions across scales rather than applying simple proportional volume reduction.

Subsequent formulation refinements replaced cyclohexane with soybean oil as the continuous phase and employed the lipophilic emulsifier polyglycerol polyricinoleate (PGPR) for droplet stabilization. PGPR, classified as Generally Recognized as Safe (GRAS) by the U.S. Food and Drug Administration (FDA),^21^ provides robust interfacial stabilization of water-in-oil nanodroplets, limiting coalescence and maintaining emulsion integrity during reaction, thereby supporting *en masse* use of hydrogel nanoparticle emulsions (HNEs) without postreaction purification.

Release kinetics in these hydrogel nanoparticles are tunable through formulation, as polymerization of different monomer ratios alters the polymeric mesh architecture. Borges, Papavasiliou, and Teymour recently extended the Flory–Rehner model to more accurately estimate multidimensional mesh dimensions in complex polymer networks (BPT extension of FR), providing a framework for therapeutic selection and diffusion-mediated release behavior design.^22,23^ This model incorporates cross-linker monomer weight and branched segments, allowing characterization of structurally complex, macromer-rich networks. Furthermore, it distinguishes between interchain distance ξ_NC_ (distance between cross-links on separate polymer chains) and intrachain distance ξ_IC_ (distance between cross-links along the same chain).^22,23^ This tunability supports applications ranging from protein to nucleic acid encapsulation and diffusive drug delivery, with multidimensional mesh parameters offering a rational framework for formulation selection based on cargo size and structure.

IPMP has been applied to biomedical nanoparticle synthesis, including systems for high-payload encapsulation, enzyme-mediated polymerization, and nucleic acid delivery.^24,25^ Within this broader framework, the present work focuses on process-scale engineering and materials efficiency while evaluating whether key measured formulation attributes are maintained after scale reduction. Across nanomaterial platforms, successful translation requires synthesis approaches that balance scalability and material efficiency with reproducible control of physicochemical properties, as variability in precursors and processing conditions can affect material structure and performance.^26^ Synthesis route and processing conditions may also influence particle size, morphology, and functional behavior, emphasizing the importance of characterizing materials attributes during process development.^27^ Accordingly, a reduced-scale synthesis method must be shown to preserve the key measurable attributes of established methods before it can be used for formulation screening and optimization.

To reduce material requirements and support formulation development with high-value biologic cargos, we developed a small-scale (15 mL) IPMP process that reduces the therapeutic cargo demand to approximately 6% of that required for bench-scale synthesis. This scale-down allows iterative optimization of formulation parameters, including network mesh architecture, while minimizing cost and resource use. Importantly, the small-scale process preserves measured formulation properties consistent with bench-scale synthesis including nanoparticle tracking analysis (NTA)-derived particle size distributions, swelling behavior, and cytocompatibility. In this study, we evaluate a small-scale IPMP process by benchmarking it against bench-scale synthesis and assess its suitability as a material-efficient strategy for optimizing nanoparticle formulations for intracellular cargo delivery.

## 2 Experimental Section

### 2.1 Materials

Poly­(ethylene glycol) diacrylate (PEGDA, *M*_w_ 575 Da, average *M*_n_ ∼509 Da by supplier NMR analysis, inhibitor <0.2%, Cat. No. 437441), n-vinyl-2-pyrrolidinone (NVP, >99% purity, Cat. No. V3409), Fluorescein *O*-acrylate (FITC *O*-acrylate, 95% purity, Cat. No. 568856) and sodium phosphate (NaH_2_PO_4_, 98.0–102.0% purity, Cat. No. S9638) were obtained from Sigma-Aldrich. PEGDA 575 was passed through a hydroquinone monomethyl ether (MEHQ) inhibitor removal column (Cat. No. 306312, Sigma-Aldrich) prior to use and was otherwise used as received. NVP was filtered through a 0.2 μm poly­(ether sulfone) (PES) filter for removal of the sodium hydroxide inhibitor. ACRL-PEG-ACRL (Mw 2000 Da, supplier-reported polydispersity indices (PDI) < 1.07), hereafter referred to as PEGDA 2000, was purchased from Laysan Bio. The thermal initiator 2,2′-azobis­(2-methylpropionamidine) dihydrochloride (V-50, 98% purity, Cat. No. 401560500) was obtained from Acros Organics. Sodium chloride (NaCl, >99.5% purity, Cat. No. BP358) was purchased from Fisher Bioreagents. Calcium Chloride (CaCl_2_, 97.0–103.0% purity, Cat. No. C69) was purchased from Fisher Chemical. Phosphate-buffered saline (PBS, pH 7.4), Dulbecco’s Modified Eagle Medium (DMEM) (Gibco, Cat. No. 31053-028), fetal bovine serum (FBS) (fisherbrand, Cat. No. FB12999102), and BODIPY 493/503 were obtained from Thermo Fisher Scientific. PGPR 4175 (purity not reported) was obtained from Palsgaard. Commercially available food-grade soybean oil was purchased from local suppliers. Acetone was obtained from Fisher Chemical. Fibroblast cell line clone 3T3 L1-MBX was purchased from the American Type Culture Collection (ATCC). Green fluorescent protein (GFP) was obtained as a laboratory-synthesized sample (see Acknowledgments).

### 2.2 Instrumentation

Emulsion processing and characterization were performed using an IPRO 250 rotor-stator homogenizer (Pro Scientific Inc.), an Ultrasonic Vibracell VCX-750 processor (SONICS), a NanoSight LM10 system (Malvern Panalytical), and a Nikon A1-HD25 confocal microscope. Instrument operating conditions are provided in the corresponding Experimental sections.

### 2.3 Emulsion Precursors and Naming Scheme

Emulsions contained tethered molecules and/or encapsulated cargo. Accordingly, formulations were denoted as *tether*-HNE­(cargo), and emulsions without either component were referred to as HNE­(blank).

Three formulations were designed for this study.

**HNE­(blank)** was an emulsion containing nanoparticles without tether or cargo. The aqueous precursor contained PEGDA 575 (0.28 M), NVP (0.12 M), V-50 (0.0069 M, equivalent to 1% of total double-bond concentration), and NaCl (0.48 M) in deionized water.

FITC**-HNE­(blank)** was an emulsion containing nanoparticles tethered with FITC but without cargo. The aqueous precursor contained PEGDA 575 (0.28 M), NVP (0.12 M), FITC *O*-acrylate (0.01 M), V-50 (0.0069 M), and NaCl (0.48 M) in deionized water.

**HNE­(GFP)** was an emulsion containing nanoparticles encapsulating GFP as cargo. The aqueous precursor contained PEGDA 2000 (0.20 M), NVP (0.20 M), V-50 (0.0060 M, equivalent to 1% of total double-bond concentration), CaCl_2_ (0.24 M), NaH_2_PO_4_ (0.24 M), and GFP (1.01 × 10^–5^ M) in deionized water.

For all formulations, the continuous phase consisted of soybean oil with 2% w/v PGPR emulsifier (Table 1).^28^

**Table 1:** Bench-Scale and Small-Scale Hydrogel Nanoparticle Formulations

	HNE(blank)	HNE(blank)	*FITC-*HNE(blank)	HNE(GFP)
**Scale**	Bench	Small	Small	Small
Ratio of Organic Phase Volume to Aqueous Phase Volume	15:1	15:1	15:1	15:1
**Organic Phase**				
**Oil**				
**Soybean Oil (mL)**	250	14	14	14
**Emulsifier**				
**PGPR (% w/v)**	2.0	2.0	2.0	2.0
**Aqueous Phase**				
**Solvent**				
**H_2_O (mL)**	17	0.93	0.93	0.93
**Monomers**				
**PEGDA 575 [M]**	0.28	0.28	0.28	0.00
**PEGDA 2000 [M]**	0.00	0.00	0.00	0.20
**NVP [M]**	0.12	0.12	0.12	0.20
**FITC *O*-acrylate [M]**	0.00	0.00	0.01	0.00
**Lipophobes**				
**NaCl [M]**	0.48	0.48	0.48	0.00
**CaCl_2_ [M]**	0.00	0.00	0.00	0.24
**NaH_2_PO_4_ [M]**	0.00	0.00	0.00	0.24
**Initiator**				
**V-50 [M]**	0.0069	0.0069	0.0069	0.0069
**Cargo**				
**GFP [M]**	0.00	0.00	0.00	1.01 × 10^‑5^

HNE­(blank) was synthesized at both bench- and small-scale, whereas FITC-HNE­(blank) and HNE­(GFP) were prepared only at small-scale. Reactions were carried out in glass reactor vessels (bench-scale) or glass scintillation vials (small-scale). The continuous-to-aqueous phase ratio was fixed at 15:1 (v/v), with formulation components scaled to the total emulsion volume.

### 2.4 Emulsion Homogenization at Bench-Scale and Small-Scale

For HNE­(blank) preparation, the aqueous phase was added dropwise (1 drop/s) into the continuous phase during homogenization at a frequency (*f*_*H*_) of 333 Hz. Emulsions were prepared at room temperature (∼20 °C) and were not precooled prior to processing. The reaction vessels were placed in an ice bath during homogenization and sonication to moderate heat accumulation. Bench-scale emulsions (267 mL) were homogenized for a duration (*t*_*H*_) of 15 min with a probe diameter (*d*_*p*_) of 20 mm (previously optimized), while small-scale emulsions (15 mL) were tested at a *t*_*H*_ of 5, 10, and 15 min with *d*_*p*_ of 7 or 20 mm, forming nonpolymerized, unreacted emulsions.

Temperature was monitored and maintained below 56 °C (the 10 h decomposition half-life of V-50)^29^ to prevent accelerated radical formation of V-50 and oxygen-mediated radical quenching.^30,31^ To identify suitable conditions for small-scale, homogenization times and probe sizes were varied, and temperature and droplet size were evaluated.

Droplet diameters were determined by NTA. Emulsion samples were diluted in cyclohexane to an effective aqueous precursor solids concentration of 0.01 mg/mL prior to analysis (preparation details provided in Supporting Information). Cyclohexane was selected to meet viscosity requirements for NTA, as Brownian motion-based measurements require low-viscosity dispersants. At 25 °C, water (a common NTA solvent) has an approximate viscosity of 1 mPa·s, cyclohexane exhibits a comparable viscosity (0.9–1 mPa·s), whereas soybean oil has a substantially higher viscosity (50–70 mPa·s), which precludes reliable particle tracking. Brief sonication was applied uniformly across samples and did not alter nanoparticle size distributions.

Homogenization parameters that produced droplet characteristics most comparable to bench-scale conditions were selected for subsequent experiments.^19^

### 2.5 Emulsion Ultrasonication at Bench-Scale and Small-Scale

New HNE­(blank) batches, homogenized under identified optimal conditions, were further processed by sonication over five cycles, each consisting of a sonication ON period (*t*_*S*_) at a 108 μm amplitude (*A*_*S*_), followed by a 30 s OFF interval (*t*_rest_). Sonication was conducted with samples maintained in an ice bath to limit temperature rise, consistent with the temperature control strategy described for homogenization. A *ts* of 1 min per cycle was used for bench-scale emulsions, while small-scale emulsions were evaluated using *t*_*S*_ periods of 5, 10, 15, and 20 s per cycle. Droplet size and emulsion temperature were recorded to identify the cycle length most comparable to bench-scale performance. The small-scale sonication parameters that produced droplet characteristics closest to bench-scale without exceeding the 10 h decomposition half-life temperature of V-50 (56 °C) were selected for subsequent experiments.

### 2.6 Emulsion Nitrogen Purging at Bench-Scale and Small-Scale

Prior to polymerization, emulsions were purged with nitrogen to reduce free-radical scavenging and promote cross-linking.^32^ Because oxygen, a free-radical scavenger, is present in air and dissolves in the soybean oil/PGPR phase, the emulsion was nitrogen purged across all conditions for reproducible processing.^33^ Bench-scale HNE­(blank) emulsions were transferred to a stirred glass reactor vessel with condensers and temperature-control baths, while small-scale emulsions remained in the glass scintillation vials (see Supporting Information for setup details). Nitrogen purging was conducted at room temperature (∼20 °C) for a purging duration (*t*_*p*_) of 45 min for bench-scale emulsions and 5 min for small-scale emulsions, with flow rates adjusted (0.2–1.0 L/min) to prevent rapid boiling. The effect of *t*_*p*_ on particle concentration was evaluated by comparing bench and small-scale emulsions using NTA.

### 2.7 Emulsion Reaction at Bench-Scale and Small-Scale

Polymerization was initiated by heating bench-scale HNE­(blank) to a temperature (*T*_*R*_) of 64 °C for a duration (*t*_*R*_) of 2.5 h under continuous stirring (*w*_*R*_ = 1.05 rad/s) and a nitrogen atmosphere (previously optimized) and by heating small-scale emulsions to a *T*_*R*_ of 60 °C in an incubator shaker (*w*_*R*_ = 7.33 rad/s), reflecting the maximum attainable temperature of the instrument (see Supporting Information for detailed setup).

To identify appropriate reaction durations, small-scale emulsions were reacted for a *t*_*R*_ of 2, 3, or 4 h and evaluated for particle diameter, particle concentration, swelling ratio, and mesh dimensions. Reaction times were selected to fall within established ranges for reproducible PEGDA/NVP polymerization behavior,^11^ and the conditions yielding particle characteristics most comparable to bench-scale outcomes were selected for subsequent studies.

After selecting the *t*_*R*_ = 2 h small-scale reaction condition for comparison with the bench-scale formulation, additional size-distribution metrics were evaluated by NTA. Reacted HNE samples were prepared for analysis using the dilution and sonication procedure described for unreacted emulsion droplets in Section 2.4 and the Supporting Information. Specifically, samples were diluted in cyclohexane to an effective aqueous precursor solids concentration of 0.01 mg/mL and briefly sonicated before analysis. The evaluated metrics included D10, D50, D90, and polydispersity index (PDI). D10, D50, and D90 were defined as the particle diameters below which 10%, 50%, and 90% of the measured particle population fell, respectively, and PDI was calculated by dividing the square of the standard deviation of the diameter by the square of the mean diameter. Each independent HNE synthesis batch was measured by NTA; particle-size distributions and distribution metrics were then summarized across independent synthesis batches, with variability reported as the standard deviation between batches.

### 2.8 Mesh Characterizations: Swelling Ratio and Mesh Estimations

Hydrogel mesh size, the spatial distance between adjacent polymer chains, was estimated using the BPT extension of FR.^22,23^

Nanoparticle swelling ratios were determined following isolation, drying, and reswelling in water (detailed postprocessing procedures provided in the Supporting Information). The swelling ratio was calculated as^28^

1 $$swelling\;ratio=\frac{swollen\;mass}{dry\;mass}$$

Mesh dimensions were determined using swelling ratio measurements and the mole fraction of the cross-linker to calculate ξ_NC_ and ξ_IC_.^22,23^

### 2.9 Emulsion Colloidal Stability

Colloidal stability of small- and bench-scale emulsions, stored at room temperature (18–22 °C) for three years, was evaluated by monitoring visible oil displacement and by NTA measurements of particle size and concentration at baseline and again after three years.

### 2.10 Emulsion Cytotoxicity Tests

To assess whether cell viability met the International Organization for Standardization (ISO) 10993-5 viability criterion of ≥70% after exposure to *en masse* emulsions and to unreacted formulation components, emulsions formulated with PEGDA 575 or PEGDA 2000 (UV sterilized) and aqueous phase constituents prepared at full formulation concentration (0% reacted) (sterilized by 0.2 μm PES filtration) were applied to 3T3 L1-MBX fibroblast clones. PEGDA 2000 (0.28 M) was included for comparison due to reported cytotoxicity associated with low-molecular-weight PEGs.^34,35^ Cells (passage 11) were cultured in 48-well plates to 70% confluency. Individual formulation components were dissolved in culture media and applied for 24 h. In contrast, *en masse* HNE formulations were applied directly for 2 h to maintain contact between the oil-continuous emulsion and the adherent cell layer, as dilution in aqueous culture media caused phase separation and reduced direct cell exposure. As this treatment required temporary removal of culture media, the HNE exposure period was limited to 2 h, after which the formulation was removed, cells were rinsed, and fresh media was restored. Cell viability was assessed 24 h after administration to capture cytotoxic effects that persisted or developed following the exposure period.

Cell viability was measured using a live/dead staining assay (Ethidium Homodimer-1 and Calcein AM; Thermo Fisher),^36^ followed by confocal imaging. Images were processed and cells were counted in ImageJ, and percent viability was calculated according to eq 2.

2 $$viable\;cells\;\left(\%\right)=\frac{live\;cell\;count}{live\;cell\;count+dead\;cell\;count}\times 100\%$$

Detailed cell handling, imaging, and analysis procedures are provided in the Supporting Information.

### 2.11 In Vitro Internalization of Nanoparticles

3T3 L1-MBX cells were differentiated into adipocytes. Cells were seeded in 48-well plates at a density of 2.73 × 10^4^ cells/cm^2^ and cultured to 60% confluency prior to induction. Differentiation was initiated using induction medium (DMEM supplemented with 10% FBS, 1% l-glutamine, 2 mg/mL dexamethasone, 0.5 mM IBMX, and 5 μg/mL porcine insulin). After 72 h, cells were transitioned to maintenance media (DMEM with 10% FBS, 1% l-glutamine, and 5 mg/mL porcine insulin) and cultured for ≥10 days to allow maturation and lipid accumulation.

Two formulations were tested.

**Formulation 1 (** *FITC* **-HNE­(blank), postprocessed):** PEGDA 575 (0.28 M), NVP (0.12 M), V-50 (1% of double bond concentration), NaCl (0.48 M), with FITC *O*-acrylate tether (0.01 M), and no cargo. Nanoparticles were purified after synthesis to remove unreacted PEGDA 575, then added to maintenance medium (0.01 mg/mL) and incubated with day-14 adipocytes for 5 min. Cells were rinsed, fixed with 4% formalin, lipid-stained with BODIPY 493/503, and imaged by confocal microscopy. Images were analyzed in ImageJ to quantify the percentage of total cellular area positive for FITC fluorescence (see Supporting Information for detailed image processing and quantification methodology).
**Formulation 2 (HNE­(GFP** **),** ***en masse*):** PEGDA 2000 (0.20 M), NVP (0.20 M), V-50 (1% of double-bond concentration), CaCl_2_ (0.24 M), NaH_2_PO_4_ (0.24 M), and GFP (1.01 × 10^–5^ M). HNE­(GFP) was prepared under sterile conditions in a biosafety cabinet using sterile reagents and equipment, diluted in sterile soybean oil (0.01 mg solids/mL) and added to day 14 adipocytes prestained with BODIPY 493/503 for 5 min. After emulsion removal, cells were imaged periodically over 8 days. PEGDA 2000 formulations were selected based on biological considerations informed by the cytotoxicity study results.

### 2.12 Statistical Analysis

Data were reported as mean ± standard deviation. Independent batches were analyzed, with technical triplicates per condition. Comparisons between two groups were evaluated using Student’s *t*-tests, while comparisons among 3 or more groups were performed using one-way or two-way ANOVA followed by Tukey’s post hoc test. In the stability test, a paired *t*-test was used for statistical analysis. Normality and homogeneity of variance were confirmed prior to parametric testing. Statistical significance was defined as *p* < 0.05; exact *p* values were provided in figure captions. The statistical analyses were conducted in GraphPad Prism 10 (GraphPad, San Diego, CA, USA).

## 3 Results and Discussion

### 3.1 Achieved Benchmarks and Success Metrics for Process Miniaturization

HNE with PEGDA 575 polymeric nanoparticles were produced at both bench-scale (267 mL) and small-scale (15 mL) using IPMP, with PGPR used to stabilize the water–oil interface.^37^ Three bench-scale emulsions served as controls, while small-scale emulsions were synthesized under varying process conditions (*d*_*p*_, *t*_*H*_, *t*_*S*_, *t*_*P*_, and *t*_*R*_).

Optimization of the small-scale process was guided by three predefined criteria:

i maintaining prereaction temperatures below 56 °C to avoid accelerated radical formation and premature polymerization
ii achieving nanoparticle diameters under 200 nm to support cellular uptake and minimize clearance, and
iii exhibiting swelling ratios of 5-6*x* relative to nanoparticle dry weight, consistent with bench-scale values.

Together, these benchmarks reflect therapeutically relevant design goals. Controlling reaction temperature prevents premature polymerization, particle diameters below 200 nm are favorable for cellular uptake, and consistent swelling ratios provide an indirect, swelling-based indicator of similar network-associated behaviors across scales. To systematically evaluate these benchmarks at small scale, we first examined homogenization conditions (Section 3.2).

### 3.2 Nanodroplet Characterization of Bench-Scale and Small-Scale after Homogenization

Bench-scale emulsions served as the benchmark for small-scale processing, and small-scale homogenization conditions were adjusted as needed to achieve comparable droplet characteristics. At bench-scale, homogenization (*d*_*p*_ = 20 mm, Figure 2A) for 15 min on ice produced stable emulsions with minimal droplet aggregation or sedimentation; however, direct translation of these parameters to small-scale resulted in emulsion instability, including visible phase separation and heterogeneous coloration, consistent with altered droplet size distributions.^28,38,39^ To mitigate these effects, the probe diameter (*d*_*p*_ = 7 mm, Figure 2B) and homogenization time (*t*_*H*_ = 5 min) were reduced. To isolate the influence of homogenization, sample volumes were collected immediately following this process step and analyzed for droplet diameter.

**Figure 2:**
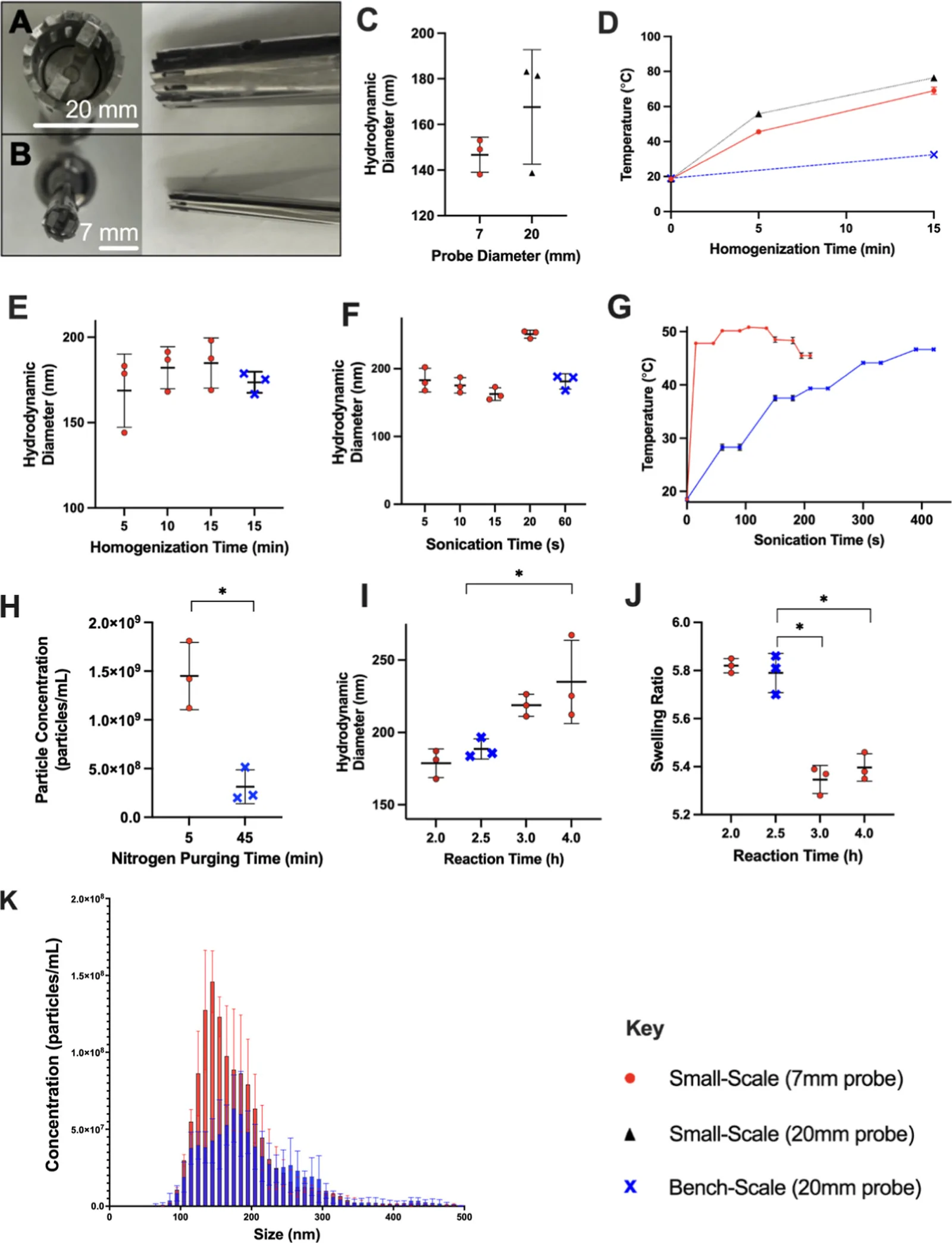
Nanoparticle diameter, emulsion temperature, and nanoparticle swelling ratios of bench-scale (**x**, blue) and small-scale (●, red or ▲, black) HNEs. (A) 20 mm homogenizer sawtooth probe. (B) 7 mm homogenizer sawtooth probe. (C) Small-scale nanodroplet diameter after homogenization with 7 mm or 20 mm probe (*n* = 3, Student’s *t*-test). (D) Hydrogel nanoparticle emulsion temperature profile during homogenization with 7 mm or 20 mm probe (*n* = 3). (E) Nanodroplet diameter after homogenization. (F) Nanodroplet diameter after five cycles of ultrasonication (*n* = 3, ordinary one-way ANOVA, *p < 0.0001). (G) Hydrogel nanoparticle emulsion temperature profile during ultrasonication (*n* = 3). (H) Nanoparticle concentration in hydrogel nanoparticle emulsions, postreaction (*n* = 3, Student's *t*-test, *p < 0.0016). (I) Nanoparticle diameter postreaction (*n* = 3). (J) Isolated nanoparticle swelling ratio (*n* = 3, ordinary one-way ANOVA, *p < 0.0001). (K) Particle size frequency distributions of bench-scale (*t*_*R*_ = 2.5 h, blue) and small-scale (*t*_*R*_ = 2.0 h, red) HNEs. Photographs in panels A and B courtesy of Ruth Negru. Copyright 2026.

Small-scale emulsions, homogenized with probe *d*_*p*_ = 20 mm, produced nanodroplets with an average diameter of 168 ± 25.1 nm (Figure 2C, *n* = 3), while those homogenized with probe *d*_*p*_ = 7 mm produced nanodroplets with an average diameter of 147 ± 7.7 nm (Figure 2C, *n* = 3). This demonstration of greater particle-size uniformity suggests the small-scale homogenization with probe *d*_*p*_ = 7 mm may produce more consistent particle sizes, which is valuable for in vitro and in vivo applications.^18,40–42^ The smaller probe also resulted in a reduced temperature spike for the small-scale emulsion (at *t*_*H*_ = 5 min, 45.5 ± 0.4 °C; at *t*_*H*_ = 15 min, 69 ± 2 °C) compared to the larger probe (at *t*_*H*_ = 5 min, 56.0 ± 1.6 °C; at *t*_*H*_ = 15 min, 76.5 ± 1.5 °C) (Figure 2D). While *t*_*H*_ between 5 and 15 min did not significantly impact particle size distribution, the temperature remained below 56 °C only with *t*_*H*_ = 5 min (Figure 2D–E). Based on this series of experiments, the optimal homogenization conditions were identified (*t*_*H*_ = 5 min, *d*_*p*_ = 7 mm). These optimized homogenization parameters consistently produced NTA-measured droplet diameters <200 nm and limited temperature rise, which supported cellular transfection requirements and reproducible cross-link formation.

### 3.3 Nanodroplet Characterization of Bench-Scale and Small-Scale after Ultrasonication

Thereafter, ultrasonication parameters were investigated.^20^ Bench-scale emulsions (*A*_*S*_ = 108 μm, *t*_*S*_ = 1 min, *t*_rest_ = 30 s) resulted in particle size distributions of 181.1 ± 11.4 nm (Figure 2F). Small-scale emulsions with reduced ultrasonication times were evaluated to limit temperature rise and thereby reduce premature thermal decomposition of V-50, which has a 10 h half-life at 56 °C but generates radicals at lower temperatures at reduced rates (*A*_*S*_ = 108 μm, *t*_*S*_ = 5, 10, 15, or 20 s, *t*_rest_ = 30 s). These sonication parameters resulted in particle diameters of 183.0 ± 17.7 nm, 175.1 ± 11.5 nm, 162.3 ± 9.5 nm, and 250.8 ± 5.8 nm, respectively (Figure 2F, *n* = 3). The period that yielded the lowest diameter and smallest size distribution (both preferable for cell interactions and replicability^43^) was *t*_*S*_ = 15 s. This parameter was further evaluated for temperature changes throughout each cycle, and the emulsion temperature did not exceed 51 °C, remaining below V-50's 10 h decomposition half-life temperature of 56 °C (Figure 2G). Optimal small-scale sonication consisted of five ultrasonication cycles (*A*_*S*_ = 108 μm, *t*_*S*_ = 15 s, *t*_rest_ = 30 s), which consistently yielded nanoparticles with NTA-derived diameters <200 nm and maintained temperatures below the initiator decomposition threshold.

### 3.4 Nanodroplet Distribution in Nitrogen-Purged Emulsions

Emulsions were purged with nitrogen to reduce the presence of oxygen, a known free-radical scavenger that can interfere with polymerization and lower nanoparticle yield.^44,45^ Although NVP copolymerization with PEGDA has been reported to mitigate oxygen inhibition through rapid propagation and early network formation that restricts oxygen diffusion,^46^ dissolved oxygen can still delay radical initiation during the induction period. To assess the effect of purging on particle concentration at small scale, postreaction samples were analyzed by NTA.

Small-scale hydrogel nanoparticle emulsion (*t*_*P*_ = 5 min, under an estimated flow rate of nitrogen ranging from 0.2–1.0 L/min, adjusted to prevent rapid boiling) contained 1.45 × 10^9^ ± 3.45 × 10^8^ particles/mL, while bench-scale hydrogel nanoparticle emulsion (*t*_*P*_ = 45 min) contained 3.14 × 10^8^ ± 1.73 × 10^8^ particles/mL (Figure 2H). Although purge duration was shorter at the small-scale, the total nitrogen volume delivered per milliliter of emulsion was greater, supporting effective nitrogen purge. Differences in particle concentration between scales are therefore attributed to scale-dependent processing effects (e.g., mixing intensity, probe geometry, and thermal gradients) rather than formulation chemistry, while the NTA-derived size distributions remained broadly comparable under the reduced nitrogen purge time frame.

### 3.5 Nanoparticle Characterization of Bench-Scale and Small-Scale Emulsions after Reaction

Emulsion reaction time (*t*_*R*_) was evaluated by comparing mean particle size, size distribution, and swelling ratios across small- and bench-scale emulsions. Although reaction temperature ramp rates differed due to inherent equipment constraints at each scale, polymerization occurred within confined aqueous droplets where radical generation and propagation kinetics are governed primarily by thermal activation rather than total batch volume. Prior kinetic modeling and gravimetric analysis of PEGDA/NVP systems demonstrated monomer conversions exceeding 85% under comparable formulation and thermal conditions.^11^ Process parameters were optimized to achieve comparable NTA-derived droplet and nanoparticle size distributions, swelling ratios, and swelling-derived mesh dimension estimations across scales. Bench-scale reactions (*t*_*R*_ = 2.5 h) resulted in particle diameters of 189 ± 7 nm (Figure 2I), while small-scale reactions (*t*_*R*_ = 2.0, 3.0, or 4.0 h) resulted in particles with progressively larger diameters of 178.7 ± 9.9 nm, 219.0 ± 7.7 nm, and 235.0 ± 28.7 nm, respectively (Figure 2I). Swelling ratios followed a similar trend; small-scale reactions (*t*_*R*_ = 2.0 h) resulted in nanoparticles with a swelling ratio (5.82 ± 0.03) which was most comparable to the swelling ratio (5.79 ± 0.08) of the bench-scale reaction (Figure 2J). Comparison of the NTA-derived particle concentration distributions within each size bin for bench-scale (*t*_*R*_ = 2.5 h) and small-scale (*t*_*R*_ = 2.0 h) HNEs showed that both formulations contained similar particle size populations across the measured size range (Figure 2K). Although the small-scale formulation exhibited a higher overall particle concentration, both formulations showed similarly shaped, overlapping size distributions across the measured range. Small-scale HNEs exhibited a D50 of 167.2 nm and a D10-D90 range of 126.8–239.6 nm. In comparison, bench-scale HNEs exhibited a D50 of 173.0 nm with a D10-D90 range of 100.1–291.9 nm, a broader but overlapping distribution. The corresponding D10, D50, D90, and PDI values are summarized with the long-term stability data in Table 2. PDI, a dimensionless measure of distribution where lower values indicate a more uniform particle size distribution, was 0.09 ± 0.01 for small-scale and 0.16 ± 0.01 for bench-scale. Consistent with these distribution metrics, both HNE formulations were primarily concentrated between 100 and 250 nm, with limited particle populations above 300 nm. Small-scale HNEs exhibited a more defined peak between 145 and 150 nm, whereas bench-scale HNEs exhibited a broader distribution extending toward larger diameters. Together, the NTA-derived distribution metrics indicated that the small-scale synthesis produced a nanoscale particle size distribution that was comparable to, and more narrowly centered than, the bench-scale formulation within the limits of NTA analysis.

**Table 2:** Particle Size Distribution Metrics of Small-Scale and Bench-Scale HNEs at *t*_0_ and *t*_3_

	Mean size (nm) ± SD	D10 (nm) ± SD	D50 (nm) ± SD	D90 (nm) ± SD	PDI ± SD
Small-scale (t_0_)	178.7 ± 9.9	126.8 ± 6.0	167.2 ± 12.5	239.6 ± 12.5	0.09 ± 0.01
Small-scale (t_3_)	184.6 ± 14.8	117.1 ± 5.0	165.5 ± 12.9	283.4 ± 26.7	0.17 ± 0.04
Bench-scale (t_0_)	189.2 ± 8.3	100.1 ± 9.1	173.0 ± 8.4	291.9 ± 4.6	0.16 ± 0.01
Bench-scale (t_3_)	179.1 ± 14.8	116.9 ± 9.8	166.0 ± 14.8	257.0 ± 28.7	0.11 ± 0.01

Mesh dimensions were calculated using the BPT extension of FR method (Figure 3)^22,23^ to estimate whether swelling-derived network-associated properties were comparable across scales. Because this model assumes network homogeneity, reported mesh dimensions were interpreted as averaged values representative of the network. Bench-scale reactions (*t*_*R*_ = 2.5 h) resulted in ξ_NC_ of 215.0 ± 2.6 Å and ξ_IC_ of 100.0 ± 2.2 Å, while small-scale reactions (*t*_*R*_ = 2, 3, or 4 h) resulted in an ξ_NC_ of 216.0 ± 0.9 Å, 201.0 ± 1.9 Å, and 203.0 ± 1.8 Å, respectively, and an ξ_IC_ of 101.0 ± 0.8 Å, 88.6 ± 1.5 Å, and 89.9 ± 1.5 Å, respectively.

**Figure 3:**
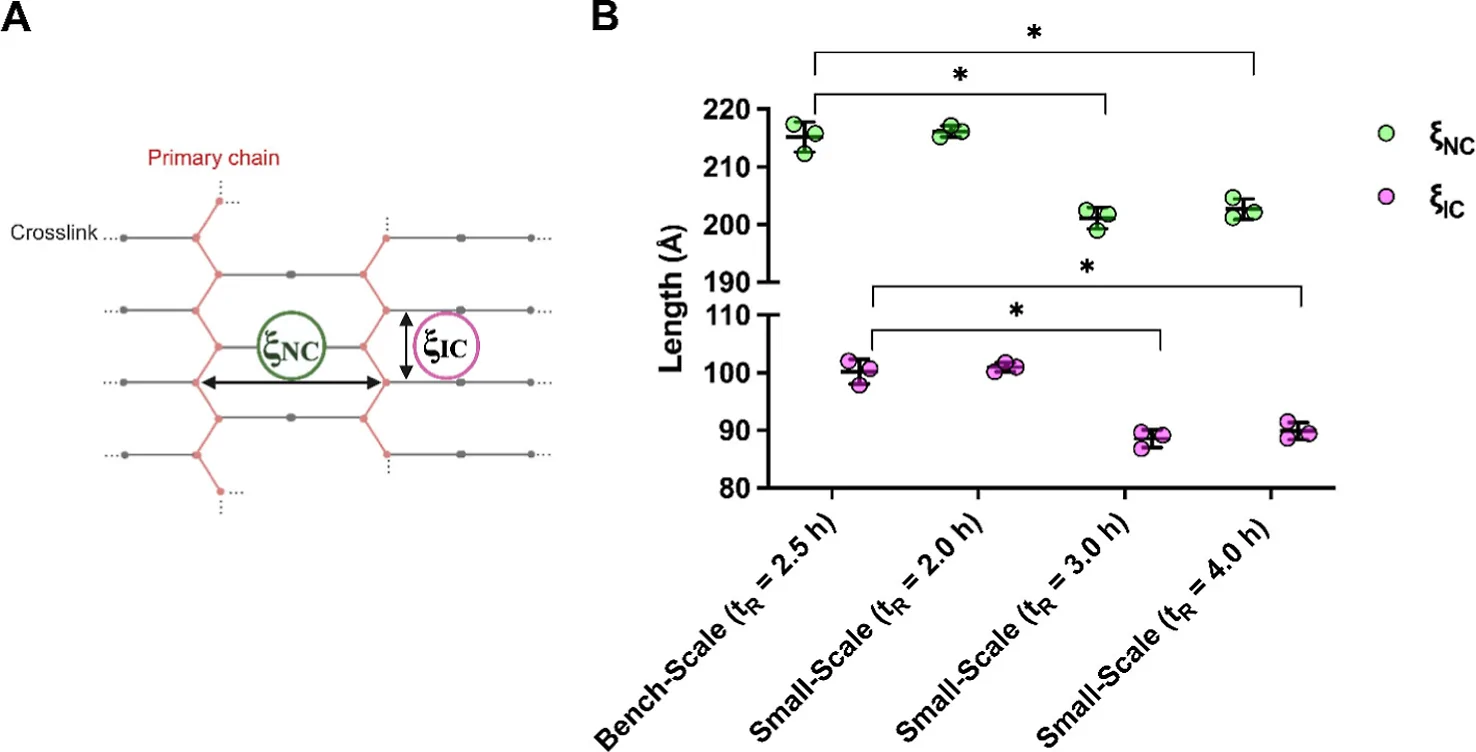
Nanoparticle interchain (ξ_NC_) and intrachain (ξ_IC_) mesh dimensions. (A) Nanoparticle dimensions according to the BPT extension of FR method. (B) ξ_NC_ (●, green) and ξ_IC_ (●, pink) dimensions of nanoparticles from bench-scale (*t*_*R*_ = 2.5 h) and small-scale (*t*_*R*_ = 2.0, 3.0, or 4.0 h) hydrogel nanoparticle emulsions (*n* = 3, ordinary two-way ANOVA, *p < 0.0001). Panel A created in BioRender. Vaicik, M. (2026) https://BioRender.com/ukt00z5.

Thus, small-scale emulsions (*t*_*R*_ = 2 h) most closely reproduce the measured network-associated properties of the bench-scale emulsions (*t*_*R*_ = 2.5 h). Although the total reaction duration differed between scales, the small-scale system reached the target temperature more rapidly due to its reduced reaction volume and faster thermal equilibration. Prior studies demonstrated high monomer consumption within approximately one hour under comparable thermal conditions,^11,47^ which supports that *t*_*R*_ = 2 h for small-scale emulsions remained sufficient to achieve high conversion. The small-scale emulsions at *t*_*R*_ = 2 h produced ξ_NC_ and ξ_IC_ values statistically indistinguishable from the bench-scale formulation (*p* > 0.05), supporting comparable swelling-derived mesh dimensions and model-estimated network properties across scales within the assumptions of the BPT extension of FR model. These mesh dimension measurements, together with comparable swelling ratios and NTA-derived particle-size distributions, support that the small-scale process maintains the evaluated formulation characteristics of the bench-scale system under the tested reaction conditions. This agreement supports the use of the small-scale process as a material-efficient platform for evaluating formulation parameters prior to subsequent scale-up.

### 3.6 Optimized Small-Scale Process Parameters Following Benchmarking

Based on these results, the final protocol for small-scale HNE synthesis was established: homogenization with *t*_*H*_ = 5 min, *f*_*H*_ = 333 Hz, *d*_*p*_ = 7 mm; ultrasonication for five cycles with *t*_*S*_ = 15 s, *A*_*S*_ = 108 μm, *t*_rest_ = 30 s; purging with nitrogen gas for *t*_*P*_ = 5 min; and reaction with *t*_*R*_ = 2 h, *T*_*R*_ = 60 °C, *w*_*R*_ = 7.33 rad/s (Figure 4). Nanoparticles synthesized under these conditions consistently met target values for NTA-derived size metrics, swelling ratio, and stability, with values comparable to those observed at bench-scale. These validated parameters were used for all subsequent emulsion cytotoxicity and application assays, with biological outcomes reflecting the optimized small-scale process.

**Figure 4:**
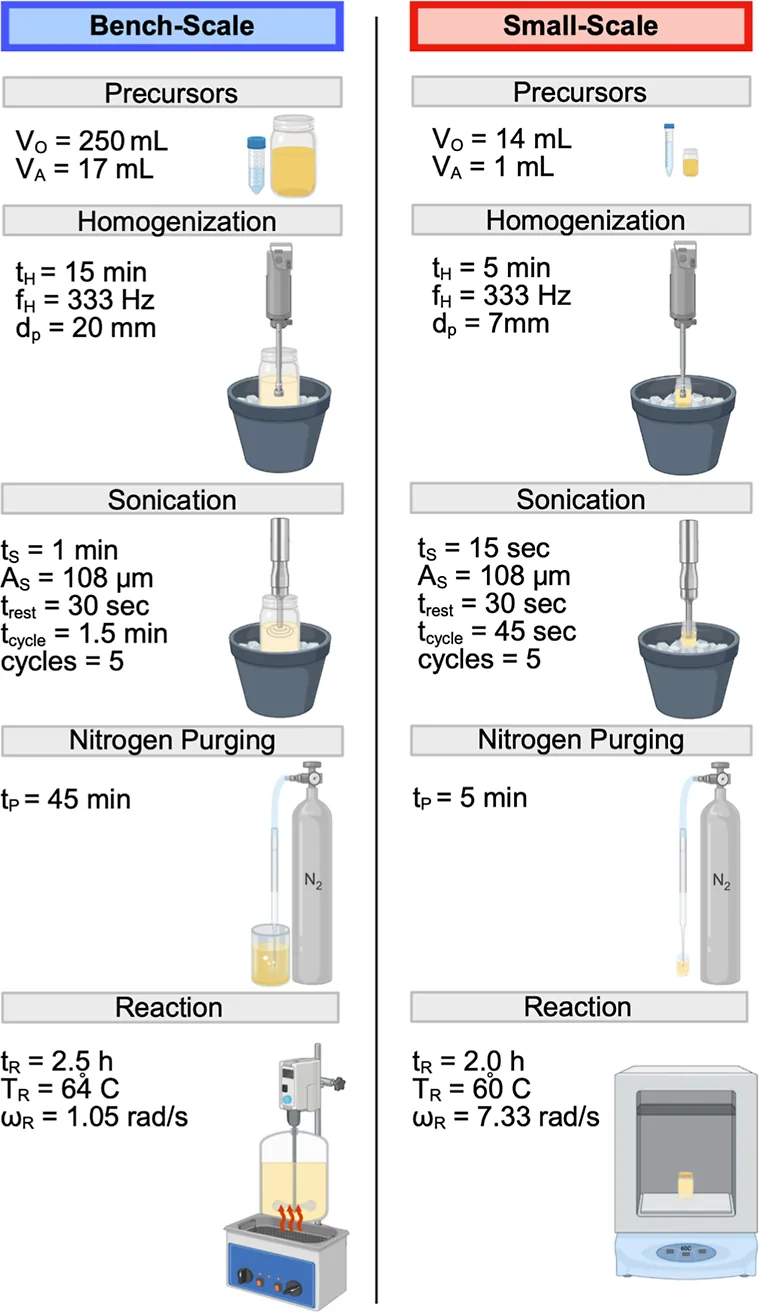
Hydrogel nanoparticle synthesis parameters for bench-scale and small-scale IPMP (*V*_*o*_ = volume of organic phase; *V*_*A*_ = volume of aqueous phase; *t*_*H*_ = homogenization time; *f*_*H*_ = homogenization frequency; *d*_*p*_ = probe diameter; *t*_*S*_ = sonication time; *A*_*S*_ = sonication amplitude; *t*_rest_ = sonication off time; *t*_cycle_ = total sonication cycle time; *t*_*P*_ = nitrogen purging time; *t*_*R*_ = reaction time; *T*_*R*_ = reaction temperature; *w*_*R*_ = angular velocity of reaction stirring). Created in BioRender. Vaicik, M. (2026) https://BioRender.com/n22x534.

### 3.7 Bench-Scale and Small-Scale Emulsion Colloidal Stability over Time

Emulsion colloidal stability, its resistance to phase separation and droplet aggregation over time, is an important parameter for therapeutic delivery systems, where extended shelf life increases translational utility.^48,49^ Surfactant and ionic salt selection strongly influence emulsion stability through reduction in interfacial tension, inhibition of droplet coalescence, suppression of Ostwald ripening by decreased droplet chemical potential.^50–52^ NaCl was used as a reference salt due to its high water solubility and prior use in this emulsion system, while CaCl_2_–NaH_2_PO_4_ at matched salt molarity was used for its cargo-stabilization relevance, looking for equivalent lipophobe function in stabilizing the emulsion.

Colloidal stability of bench-scale and small-scale emulsions was assessed after storage for three years at room temperature (18–22 °C) (Figure 5A,B). Both small- and bench-scale HNE­(blank) retained stability after three years postsynthesis (*t*_3_). Neither showed significant changes in mean particle size (small-scale: *t*_0_ = 178.7 ± 9.9 nm, *t*_3_ = 184.6 ± 14.8 nm; bench-scale: *t*_0_ = 189.2 ± 8.3 nm, *t*_3_ = 179.1 ± 14.8 nm, Figure 5C, Table 2) or concentration (small-scale: *t*_0_ = 1.45 × 10^9^ ± 3.46 × 10^8^ nanoparticles/mL, *t*_3_ = 1.70 × 10^9^ ± 4.45 × 10^8^ nanoparticles/mL; bench-scale: *t*_0_ = 3.14 × 10^8^ ± 1.73 × 10^8^ nanoparticles/mL, *t*_3_ = 5.59 × 10^8^ ± 7.31 × 10^7^ nanoparticles/mL, Figure 5D). D50 values also remained closely aligned across storage time (small-scale: 167.2 ± 12.5 nm at *t*_0_ and 165.5 ± 12.9 nm at *t*_3_; bench-scale: 173.0 ± 8.4 nm at *t*_0_ and 166.0 ± 14.8 nm at *t*_3_), indicating that the median particle populations were preserved over three years (Table 2). D10 and D90 values further indicated that the central NTA-detected particle populations remained within the nanoscale range. PDI values remained low across all formulations and time points, ranging from 0.09 ± 0.01 to 0.17 ± 0.04, supporting relatively narrow particle size distributions.

**Figure 5:**
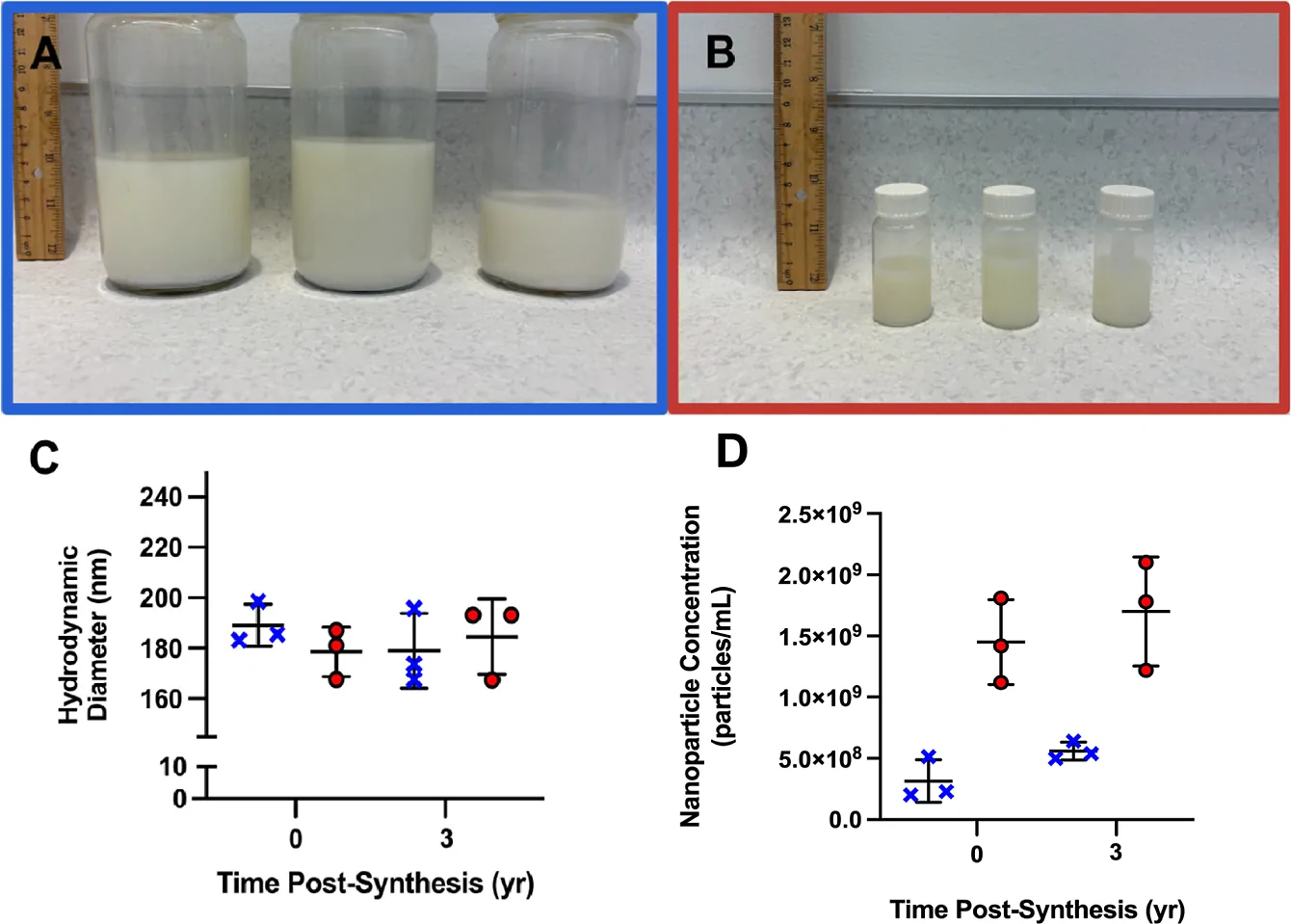
Colloidal stability of bench-scale and small-scale hydrogel nanoparticle emulsions. Images of hydrogel nanoparticle emulsions formed using (A) bench-scale (blue) and (B) small-scale (red), demonstrating colloidal stability at 18–22 °C for three years postsynthesis. (C) Hydrodynamic diameters (*n* = 3, paired *t*-test) and (D) nanoparticle concentration (*n* = 3, paired *t*-test) of hydrogel nanoparticle emulsions formed using bench-scale (x, blue) and small-scale (●, red), demonstrating colloidal stability at 18–22 °C for three years postsynthesis. Photographs in panels A and B courtesy of Ruth Negru. Copyright 2026.

Together, these results indicated that HNEs prepared at both bench- and small-scale retained their dominant nanoscale particle populations over three years, with no significant shifts in mean size or median diameter. These findings support the physical colloidal stability of the emulsions during extended storage and suggest that small-scale synthesis can produce HNEs comparable to bench-scale HNEs with respect to the measured colloidal stability and NTA-derived size metrics.

### 3.8 In Vitro Cytocompatibility of Emulsions and Precursor Components

*En masse* emulsions offered advantages for hydrophilic drug delivery, such as reduced therapeutic loss due to purification, mitigation of drug burst release associated with nanoparticle drying, and protection against cargo degradation.^53–55^ However, direct exposure to unreacted formulation precursors or leachable components may pose cytotoxic risk, particularly for lower-molecular-weight PEGDA formulations. To evaluate this potential risk, emulsions formulated with PEGDA 575 or PEGDA 2000, along with individual aqueous-phase components at full formulation concentration (0% reacted), were assessed in 3T3 L1-MBX fibroblast clones, with viability normalized to untreated controls (Figure 6). Given reports of increased cytotoxicity associated with lower-molecular-weight PEGs, PEGDA 2000 (0.28 M) was included as a higher-molecular-weight comparison.^34,35^

**Figure 6:**
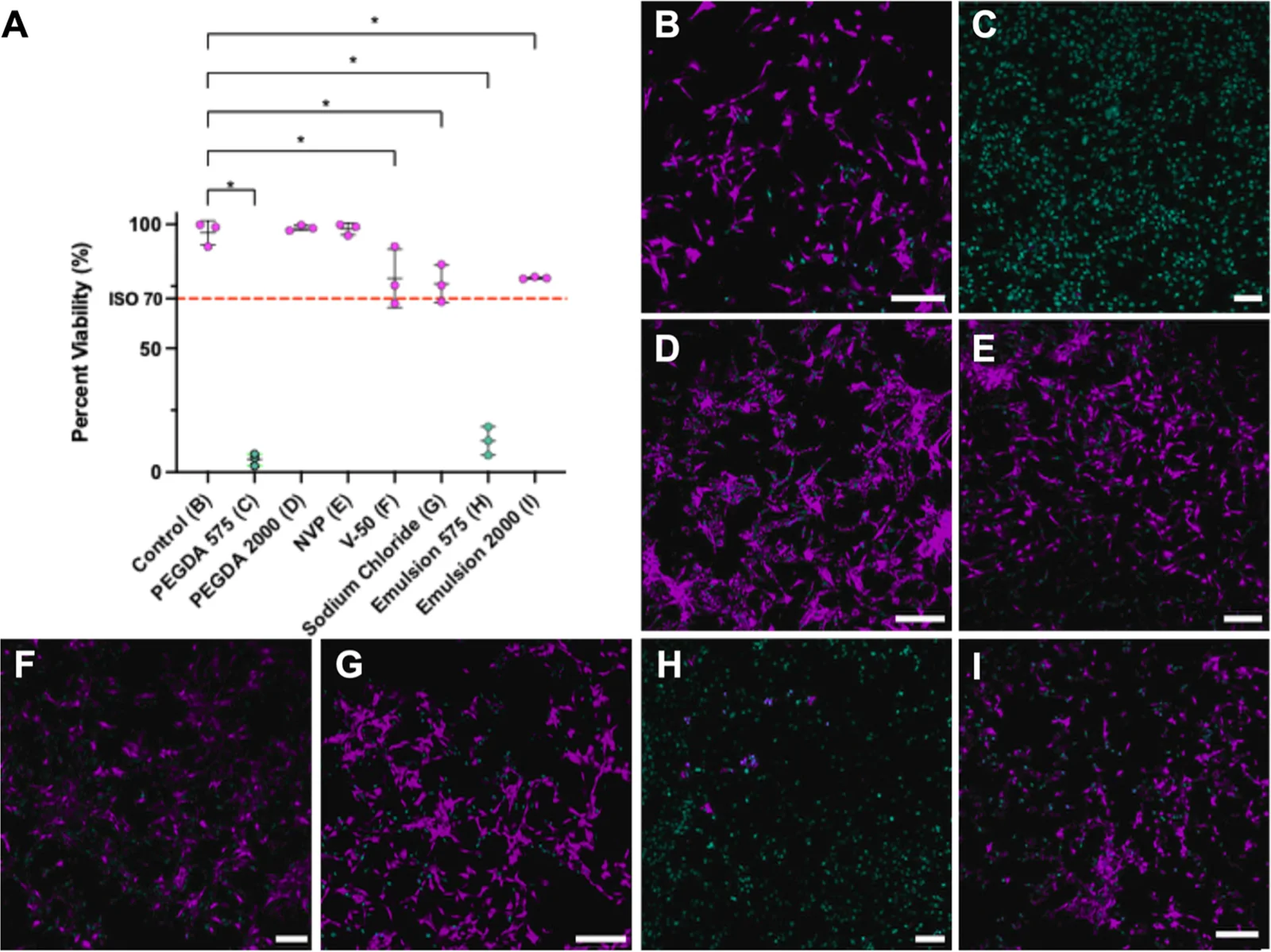
In vitro viability of 3T3 L1 preadipocytes 24 h after exposure to HNE­(blank) precursor components and *en masse* emulsions. (A) Percent viability of cells following exposure to HNE­(blank) and precursor components relative to the ISO 10993-5 cytotoxicity threshold (≥70% viability; red dotted line). (B–I) 3T3 L1 cells stained with Live/Dead Calcein-AM (live, purple) and Ethidium Homodimer-1 (dead, teal) after exposure to (B) control growth media, (C) PEGDA 575, (D) PEGDA 2000, (E) NVP, (F) V-50, (G) Sodium Chloride, (H) *en masse* HNE­(blank) formulated with PEGDA 575, and (I) *en masse* HNE­(blank) formulated with PEGDA 2000 (*n* = 3, ordinary one-way ANOVA, *p < 0.05). Scale bar = 20 μm. Live/Dead colors were adjusted for color-deficiency accessibility (green/red to purple/teal).

Quantitative viability results were summarized in Figure 6A, and representative images (Figure 6B–I) were visually consistent with these results. The untreated control (Figure 6B) had near-complete viability (96.6 ± 4.9%). Cells treated with free PEGDA 575 (Figure 6C) exhibited the lowest viability (5.19 ± 2.41%), demonstrating poor tolerance of the unreacted low-molecular-weight precursor at the tested concentration. PEGDA 575-based HNEs also produced low viability (Figure 6H, 12.7 ± 5.7%), indicating that polymerization into the emulsion formulation did not sufficiently reduce cytotoxicity under the tested conditions. In contrast, cells treated with free PEGDA 2000 (Figure 6D) maintained high viability (98.6 ± 1.1%), as did those treated with NVP (Figure 6E, 98.1 ± 2.3%), the initiator V-50 (Figure 6F, 78.2 ± 11.9%), and sodium chloride (Figure 6G, 76.0 ± 7.6%). Emulsions formulated with PEGDA 2000 (Figure 6I) also maintained high viability (78.3 ± 0.4%) and exceeded the ISO 10993-5 cytotoxicity threshold (≥70% viability), indicating cytocompatibility under the tested conditions. Although the specific contribution of unreacted PEGDA, soluble oligomers, or other leachable components was not quantified, the marked difference between PEGDA 575- and PEGDA 2000-based formulations indicates that PEGDA molecular weight strongly influences cytocompatibility. Because soybean oil and PGPR constitute the continuous phase presented to cells, cytotoxicity was evaluated for the complete emulsion system.

Collectively, these results demonstrate that PEGDA 2000-based HNEs can be administered without postprocessing or phase separation while maintaining viability above the ISO 10993-5 cytotoxicity threshold under the tested conditions. In contrast, PEGDA 575-based HNEs may require reduced application, purification, or reformulation before direct biological application. These findings represent acute, screening-level cytocompatibility and identify PEGDA molecular weight as a critical formulation parameter. The substantial improvement observed with PEGDA 2000 supports selection of higher-molecular-weight formulations for direct *en masse* administration, whereas PEGDA 575-based formulations may require purification or reformulation before biological use.

### 3.9 Cellular Uptake and Visualization of Fluorescent Nanoparticles

3T3 L1-MBX cells were differentiated into adipocytes to assess nanoparticle uptake in a lipid-rich, low-activity cell model.^56–59^ We evaluated the uptake of extracted FITC-HNE­(blank) nanoparticles, diameter 175.9 ± 2.2 nm (*n* = 1), in differentiated adipocyte cells (Figure 7, *n* = 4). Untreated adipocytes and adipocytes exposed to extracted HNE­(blank) nanoparticles were imaged as negative controls.

**Figure 7:**
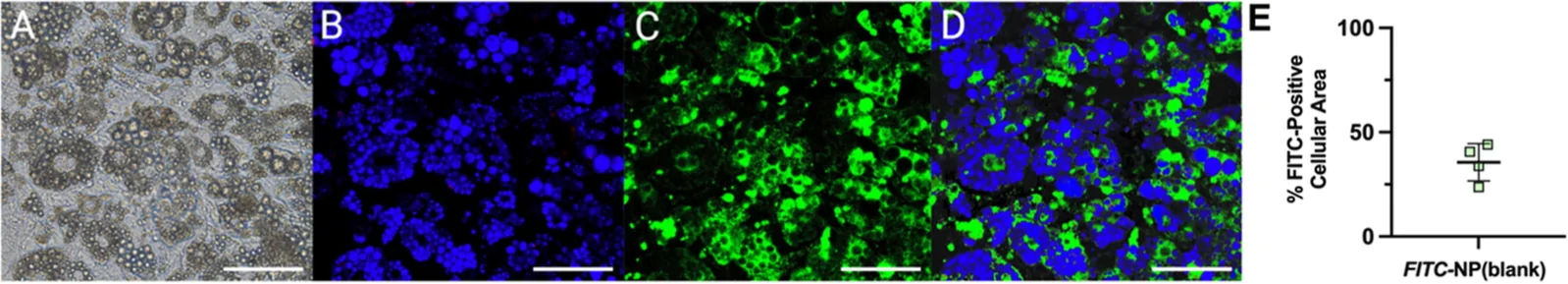
Intracellular accumulation of FITC-labeled nanoparticles in differentiated 3T3 L1 in vitro. (A) Brightfield image of the differentiated 3T3 L1 cells, noting high lipid content, (B) BODIPY lipid (blue), (C) FITC*-*nanoparticles (green), (D) fluorescence overlay of intracellular FITC*-*nanoparticles (green) and BODIPY-stained lipids (blue), and (E) quantification of FITC-nanoparticle (blank) uptake in 3T3 L1 monolayer (noncellular background excluded), mean ± SD (*n* = 4). Scale bar = 50 μm. (Colors were adjusted for color-deficiency viewing from Green/Red to Green/Blue).

Bright-field imaging (Figure 7A) confirmed the morphology of differentiated adipocytes, noting large, coalesced lipid droplets. Signals for lipid and FITC-nanoparticles were displayed separately in Figure 7B,C, respectively, and fluorescent overlays (Figure 7D) showed differentiated adipocytes with both stained lipid droplets (blue) and an accumulation of FITC*-*nanoparticles (green). Imaging quantification indicated that 35.6 ± 9.0% of total cellular area was FITC*-*nanoparticle-positive (Figure 7E). Notably, differentiated adipocytes contained 64.4 ± 9.0% lipid area, which is not available for nanoparticle internalization. When considered relative to the nonlipid cytoplasmic fraction, FITC*-*nanoparticle signal occupied the majority of accessible intracellular space, while control groups exhibited no detectable fluorescence, confirming specificity of nanoparticle-associated signal. Despite typical adipocyte resistance to transfection,^56–58^ FITC*-*nanoparticles efficiently crossed the cell membrane and localized in the cytoplasm, consistent with nanoparticle uptake and intracellular retention in adipocytes.

### 3.10 En Masse HNE Administration Results in Nanoparticle Internalization and Delayed Intracellular GFP-Associated Fluorescence

Nanoparticle internalization and the time-dependent appearance of GFP-associated fluorescence were evaluated in differentiated adipocytes using *en masse* HNE­(GFP) produced with the previously validated small-scale synthesis process (Figure 8A–C). The HNE­(GFP) batch used for this functional experiment had a mean particle diameter of 171.4 ± 3.1 nm, ξ_NC_ 356.5 ± 7.2 Å, and ξ_IC_ 55.6 ± 5.6 Å (N = 1 synthesis batch, *n* = 3 technical measurements from separate aliquots of the same batch). These measurements characterized the formulation administered in the cell study and were not intended to assess batch-to-batch synthesis reproducibility. Untreated adipocytes and adipocytes exposed to HNE­(blank) were imaged as negative controls.

**Figure 8:**
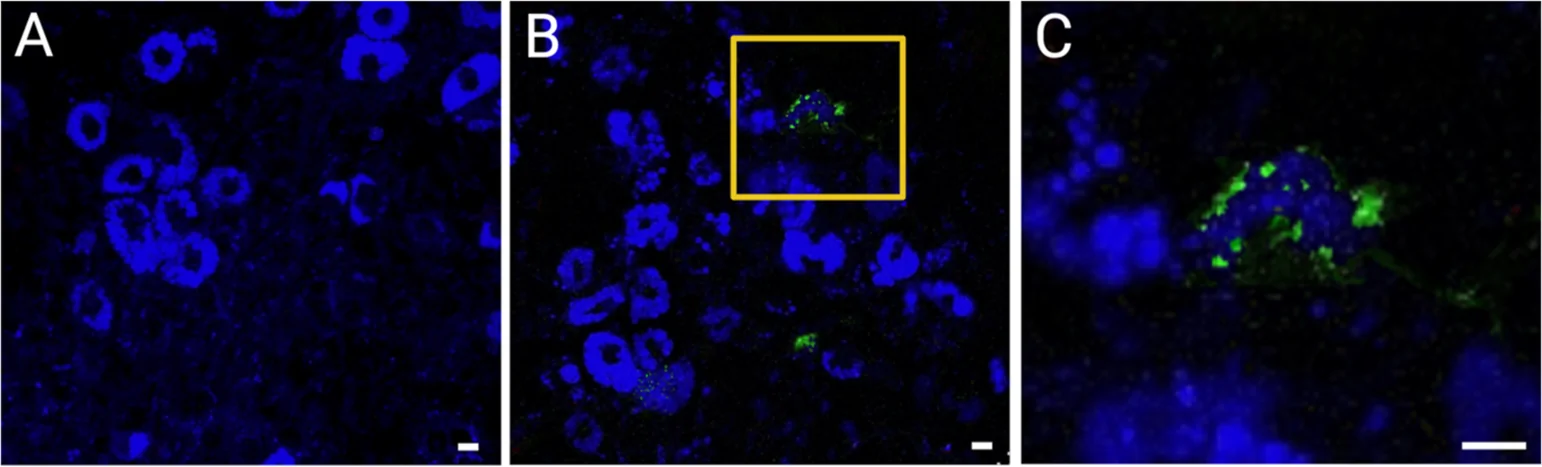
Differentiated 3T3 L1 in vitro application of HNE­(GFP) and intracellular GFP-associated fluorescence over 8 days. (A) Fluorescence overlay of HNE­(GFP) diffusive-release (green) and BODIPY-stained lipids (blue) 1 day after emulsion dosing, (B) fluorescence overlay of GFP-associated release (green) and BODIPY-stained lipids (blue) 8 days after emulsion dosing, and (C) magnification of GFP-associated release 8 days after emulsion dosing (yellow box). Scale bar = 10 μm. (Colors were adjusted for color-deficiency viewing from Green/Red to Green/Blue).

Rapid nanoparticle internalization was confirmed using fluorescently tagged FITC*-*HNE­(blank). Neither untreated adipocytes nor adipocytes treated with HNE­(blank) exhibited detectable fluorescence. In adipocytes treated with untagged HNE­(GFP), no detectable GFP-associated signal was observed 1 day after HNE­(GFP) application (Figure 8A), whereas cytoplasmic GFP-associated fluorescence was evident by day 8 in a subset of cells imaged within the same cellular region (Figure 8B,C).

Because extracellular HNE was removed following administration and nanoparticle internalization had already occurred, the delayed appearance and cytoplasmic distribution of GFP-associated fluorescence are consistent with time-dependent intracellular release of GFP cargo. These qualitative imaging data distinguish GFP-associated signal from the immediate nanoparticle internalization demonstrated using FITC-labeled nanoparticles. Accordingly, the delayed appearance of GFP-associated fluorescence was interpreted as qualitative evidence of time-dependent intracellular cargo release and as a functional demonstration that the small-scale HNE platform can support intracellular cargo delivery.

The detection of GFP-associated fluorescence at day 8 indicates that the cargo retained fluorescence following HNE synthesis, intracellular delivery, and release from the nanoparticle formulation. Although the present imaging study was not designed to quantify cumulative release kinetics, the temporal pattern of GFP-associated fluorescence provides additional evidence that HNE produced using the small-scale process can maintain the fluorescence of cargo and may support its delayed intracellular availability.

### 3.11 Enhancing Biologic Delivery through Scalable Hydrogel Nanoparticle Emulsion Platforms

Polymer-based hydrogels have been widely investigated as drug-delivery platforms because their hydrated, tunable networks can accommodate diverse cargos, support controlled release, and be functionalized for specific delivery applications^60^ through properties including polymer composition, cross-linking, swelling, and mesh structure.^49^ Within this broader class of materials, IPMP has emerged as a promising strategy for synthesizing hydrogel nanoparticles for therapeutic delivery; however, many reported systems require large reaction volumes, multiple surfactants, or extensive postprocessing steps that complicate reproducibility, increase material consumption, and limit efficient optimization when working with high-value biologic cargos.^61^ These limitations create a barrier to rapid formulation development and translation of nanoparticle delivery platforms.

In this study, we demonstrate that controlled small-volume IPMP synthesis can reproducibly generate PEGDA-NVP hydrogel nanoparticles with measured formulation and performance characteristics comparable to those produced at bench-scale. This scale reduction is consistent with the droplet-confined nature of IPMP, in which radical polymerization occurs within stabilized aqueous nanodroplets dispersed in a continuous oil phase. Because polymerization proceeds within these localized reaction environments, bulk reactor volume may have a limited influence on particle formation when key processing conditions are preserved. Accordingly, comparable nanoparticle size distributions, swelling behavior, and swelling-derived mesh characteristics were observed across scales. The use of multiple complementary measurements is consistent with prior polymeric biomaterial studies, including work on chitosan-alginate-carrageenan composite films in which swelling behavior was evaluated together with solubility, mechanical properties, and antibacterial performance to relate hydration behavior to broader material function.^25,62^ Together, these findings support evaluation of the small-scale process through a coordinated set of particle and network-associated properties rather than reliance on any single characterization metric.

Compared to many reported IPMP systems that rely on multiple surfactants and experience challenges with postextraction aggregation,^61^ the formulation used here employs a single surfactant (PGPR) to limit coalescence, reducing formulation complexity while supporting reproducibility and scalability. Importantly, nanoparticle stability within the emulsion phase allows the formulations to be administered directly without nanoparticle extraction or postprocessing, reducing material loss and simplifying preparation for biologic delivery applications. The PEGDA/NVP hydrogel nanoparticles described in this work exhibit rapid adipocyte internalization (Figures 7 and 8), delayed intracellular GFP-associated fluorescence consistent with time-dependent cargo release (Figure 8), colloidal stability over three years at room temperature (Figure 5), and cytocompatibility of the tested formulations and precursor concentrations (Figure 6).

In comparison, poly­(ethylenimine) (PEI), although widely used for nucleic acid delivery due to its high transfection efficiency and cargo protection, presents reported challenges including cytotoxicity, rapid clearance, and limited targeting specificity.^63^ PEGylated PEIs and cleavable PEG linker strategies have been explored to mitigate these limitations but introduce additional formulation complexity.^64^ In contrast, the PEG chemistry used in the present system provides straightforward ligand functionalization while maintaining formulation simplicity and colloidal stability.

This study establishes an empirically validated framework for small-scale IPMP synthesis rather than a generalized scaling law. Comparable NTA-derived particle size distributions, swelling behavior, swelling-derived mesh dimensions, colloidal stability, and cellular performance were observed under matched processing conditions. However, dimensionless scaling relationships governing droplet formation and energy input were not explicitly derived. Furthermore, because particle size and dispersity were evaluated by NTA without orthogonal confirmation, the present findings should be interpreted as comparability in the evaluated formulation attributes rather than complete confirmation of nanoparticle equivalence across scales. Cargo release was evaluated qualitatively in an intracellular context, and quantitative release kinetics were not resolved. Surface charge was not directly determined for the *en masse* HNE because conventional zeta-potential analysis would require disruption of the native PGPR-stabilized W/O formulation.^65^ Accordingly, the present study defines the small-scale HNE process and its ability to maintain evaluated formulation properties and support intracellular delivery behavior. Cargo-specific loading, quantitative release kinetics, and in vivo performance constitute separate formulation and translational objectives.

Collectively, these findings position the HNE platform as a material-efficient, scale-down-compatible system for intracellular biologic cargo delivery. Reproducible synthesis across bench and small scales preserved nanoparticle properties and cytocompatibility, while direct administration of *en masse* emulsions reduced material loss and eliminated the need for particle extraction. Together, these advantages make the small-scale process well suited for iterative formulation development with high-value protein and nucleic acid cargos.

All raw data underlying the figures and analyses, supporting the conclusions of this work, are available in the data repository (see Associated Content).^66^

## 4 Conclusions

A small-scale (15 mL) IPMP process was developed that reduced material requirements by >90% compared to bench-scale synthesis while preserving NTA-derived nanoparticle size metrics, swelling ratios, swelling-derived mesh dimensions, and colloidal stability. The optimized small-scale protocol identified critical process parameters for homogenization, sonication, nitrogen purging, and reaction time, producing HNEs with reproducible measured formulation properties. Cytotoxicity testing demonstrated that PEGDA-2000 formulations maintained cell viability above ISO 10993-5 cytotoxicity threshold (≥70% viability). *En masse* emulsion administration resulted in rapid nanoparticle uptake and intracellular GFP-associated fluorescence by day 8 in adipocytes, consistent with time-dependent cargo release. These findings support comparability between the small-scale and bench-scale processes in the evaluated attributes and provide an efficient platform for iterative optimization of formulation parameters before subsequent scale-up.

## Supplementary Material



## Notes

Standard green/red images for nanoparticle emulsion cytotoxicity studies, FITC-nanoparticle administration into adipocytes, and HNE­(GFP) application in adipocytes with GFP released (PDF)

## Abbreviations

ATCC: american type culture collection
BPT extension of FR: borges-papavasiliou-teymour extension of flory–rehner
DMEM: dulbecco’s modified eagle medium
FBS: fetal bovine serum
FDA: food and drug administration
FITC *O*-acrylate: fluorescein *O*-acrylate
GFP: green fluorescent protein
GRAS: generally-recognized-as-safe
HNE: hydrogel nanoparticle emulsion
IBMX: 3-Isobutyl-1-methylxanthine
IPMP: inverse phase miniemulsion polymerization
ISO: international organization for standardization
MEHQ: hydroquinone monomethyl ether
MeSH: medical subject headings
NaCl: sodium chloride
NVP: *n*-vinyl-2-pyrrolidinone
PBS: phosphate-buffered saline
PEG: poly­(ethylene glycol)
PEGDA: poly­(ethylene glycol) diacrylate
PEI: poly­(ethylenimine)
PES: polyethersulfone
PGPR: polyglycerol polyricinoleate
VEGF: vascular endothelial growth factor
V-50: 2,2’ -azobis­(2-methylpropionamidine) dihydrochloride
W/O: water in oil
3T3 L1: 3T3 L1-MBX preadipocytes.
