# Supplementary material for: Process Design for Small-Scale Production of PEGDA-NVP Hydrogel Nanoparticle Emulsions for Therapeutic Applications
Source: ACS Omega. 2026 Aug 12;11(43):66317–30. doi: 10.1021/acsomega.6c04988 (PMC13750379; doi:10.1021/acsomega.6c04988)
Supplement: Supplementary file 1 [file ao6c04988_si_001.pdf]

Supporting Information

Process Design for Small-Scale Production of PEGDA-NVP Hydrogel Nanoparticle Emulsions for Therapeutic Applications

Ruth Negru[1], *Fernando T.P. Borges*[2], *Georgia Papavasiliou*[2], *Fouad Teymour*[2], *Marcella K. Vaicik*[1,*]

[1]Department of Biomedical Engineering, Illinois Institute of Technology, Chicago, IL

[2]Department of Chemical and Biological Engineering, Illinois Institute of Technology, Chicago, IL

* Corresponding Author

Email*: mvaicik@illinoistech.edu

S1. Supplementary Methods

The following sections provide additional procedural details that support the experimental methods described in the main manuscript. These descriptions are included to facilitate reproducibility.

Nanoparticle Tracking Analysis (NTA). Droplet size distributions were measured using a NanoSight LM10 (Malvern Panalytical). For each formulation, an aliquot that corresponded to approximately 1 mg of aqueous precursor solids (PEGDA, NVP, salts, and cargo), calculated from the initial weighed formulation inputs, was drawn for analysis. The withdrawal volume was adjusted by proportion to account for formulation-dependent differences in molecular weight, salt content, and cargo content.

The withdrawn aliquot was diluted in 1 mL of cyclohexane and sonicated for 10 s. A secondary dilution was then performed by transferring 10 µL of the suspension into 990 µL of cyclohexane to achieve a final concentration of approximately 0.01 mg/mL, which reproducibly yielded particle concentrations within the recommended range for NTA acquisition (~200-500 particles per frame). Samples were briefly sonicated prior to measurement to distribute the aliquot into the solvent. Particle size distributions and concentrations were quantified by NTA assessment of particle light scattering and Brownian motion.

Nitrogen Purging and Reaction Setup. For bench-scale HNE synthesis, emulsions were transferred to a stirred glass reactor vessel equipped with condensers and temperature control baths. Small-scale emulsions were processed in 20 mL glass scintillation vials sealed with parafilm, which was punctured to allow nitrogen introduction via pipette (Figure S1).

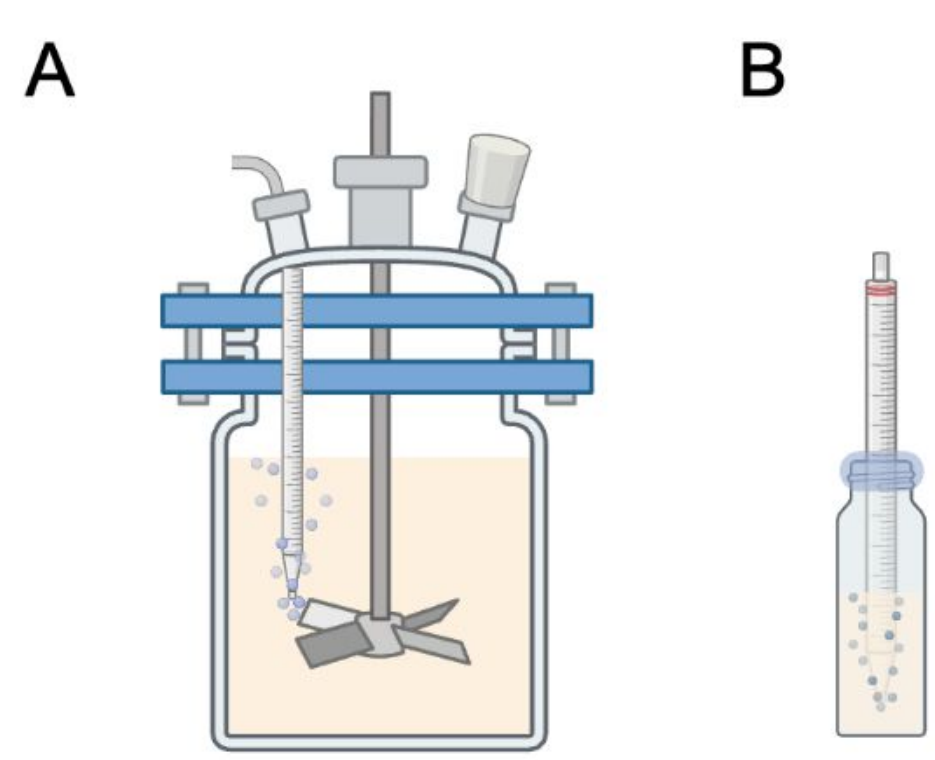

Figure S1. Schematic of nitrogen purging setups for (A) bench-scale and (B) small-scale inverse phase miniemulsion polymerization. Created in BioRender. Vaicik, M. (2026) https://BioRender.com/l74m136.

Bench-scale reactions were conducted in the stirred glass reactor vessel, immersed in an oil bath, and connected to a recirculating cooling system, while small-scale reactions took place within sealed scintillation vials placed inside an incubator shaker (Figure S2).

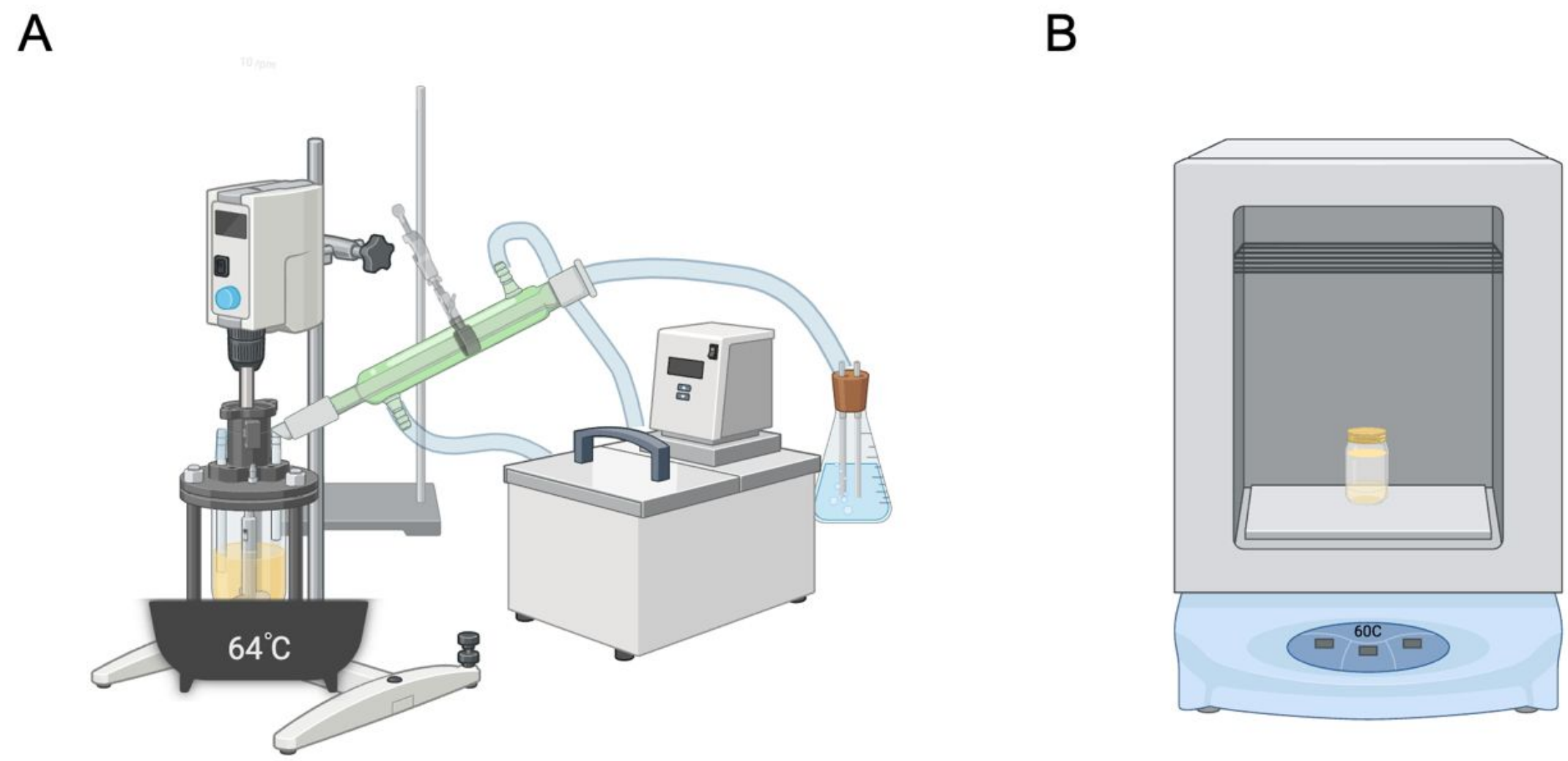

Figure S2. Schematic of (A) bench-scale and (B) small-scale IPMP. Created in BioRender. Vaicik, M. (2026) https://BioRender.com/hjlsff0.

Nanoparticle Isolation and Swelling Ratio Determination. To determine the particle swelling ratio, emulsions were first post-processed to isolate nanoparticles from the organic phase. Acetone (equivalent to 60% of the total emulsion volume) was added to induce particle precipitation, and samples were stored at 4 °C overnight to allow phase separation. The oil and acetone phases were removed, and the particles were rinsed with fresh acetone and centrifuged for 30 min (3200 x g, 4 °C). The acetone and centrifugation steps were repeated 2-3 times until the oil phase was washed away. Isolated nanoparticles were dried in a vacuum oven for 48 h at 40 °C and weighed to obtain their dry mass. Dried particles were then resuspended in water and allowed to swell for 72 h. After centrifugation to remove excess supernatant, the swollen mass was recorded. The ratio of swollen to dry weight was recorded.

Live/Dead Cytotoxicity Analysis. Images obtained from confocal microscopy after live/dead staining were processed separately in ImageJ. Individual channels for live and dead cells were converted to 8-bit grayscale, thresholded to generate binary masks, and analyzed using the function “Analyze Particles” to quantify cell counts. Viability was calculated as the ratio of live cells to total cells.

Quantification of FITC-Positive Cellular Area in Adipocytes. Confocal images were analyzed in ImageJ to quantify fluorescence-positive area. Color images were first separated using the "Color Split" function to isolate individual fluorescence channels. Spatial calibration was performed by converting pixels to micrometers using the scale bar embedded in each image. Images were auto-thresholded to define fluorescence-positive signal, and only pixels within manually defined cellular region of interest were included in the analysis. The number of threshold-positive pixels for each channel (FITC and BODIPY) was quantified and converted to area measurements (cm^2) based on the calibrated pixel-to-length conversion. FITC-positive area was reported in reference to the total cellular area within the image and the plated well area.

S2. Standard Fluorescence Color Representations

The confocal microscopy images presented here (Figures S3-S5) in standard red/green fluorescence coloration correspond to Figures 6-8 in the main manuscript, respectively.

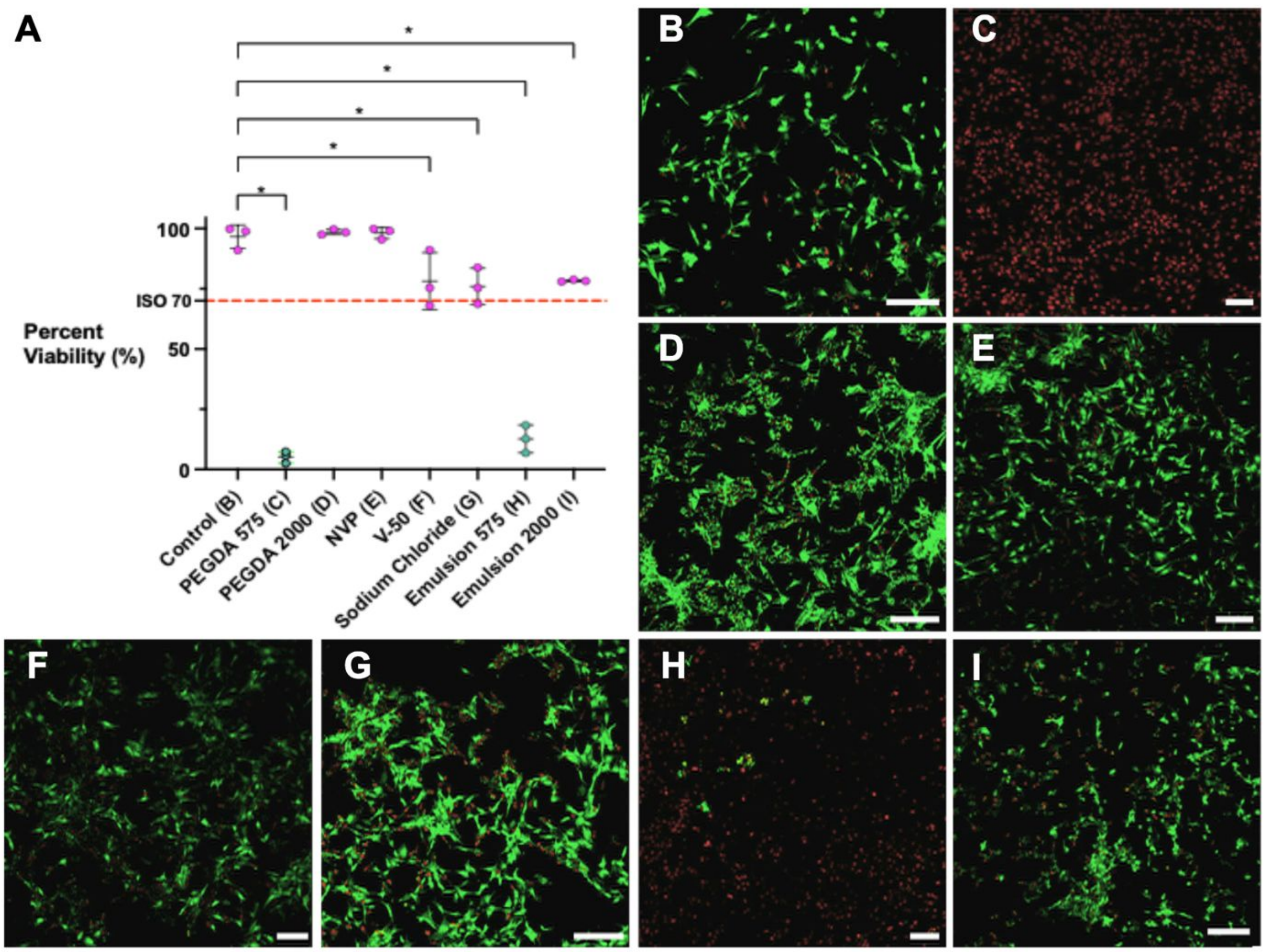

Figure S3. In vitro viability of 3T3 L1 preadipocytes 24 hours after exposure to HNE(blank) precursor components and *en masse* emulsions. (A) Percent viability of cells after exposure to HNE(blank) and precursor components, red dashed line indicates the cytotoxicity threshold at 70% for the International Organization for Standardization (ISO-standard). (B-I) 3T3 L1 cells stained with Live/Dead Calcein-AM (**green** - live) and Ethidium Homodimer-1 (**red** - dead) after exposure to (B) control standard growth media, (C) PEGDA 575, (D) PEGDA 2000, (E) NVP, (F) V-50, (G) Sodium Chloride, (H) en masse HNE(blank) formulated with PEGDA 575, and (I) *en masse* HNE(blank) formulated with PEGDA 2000 (n=3, ordinary one-way ANOVA, *p<0.05). Scale bar = 20 μm.

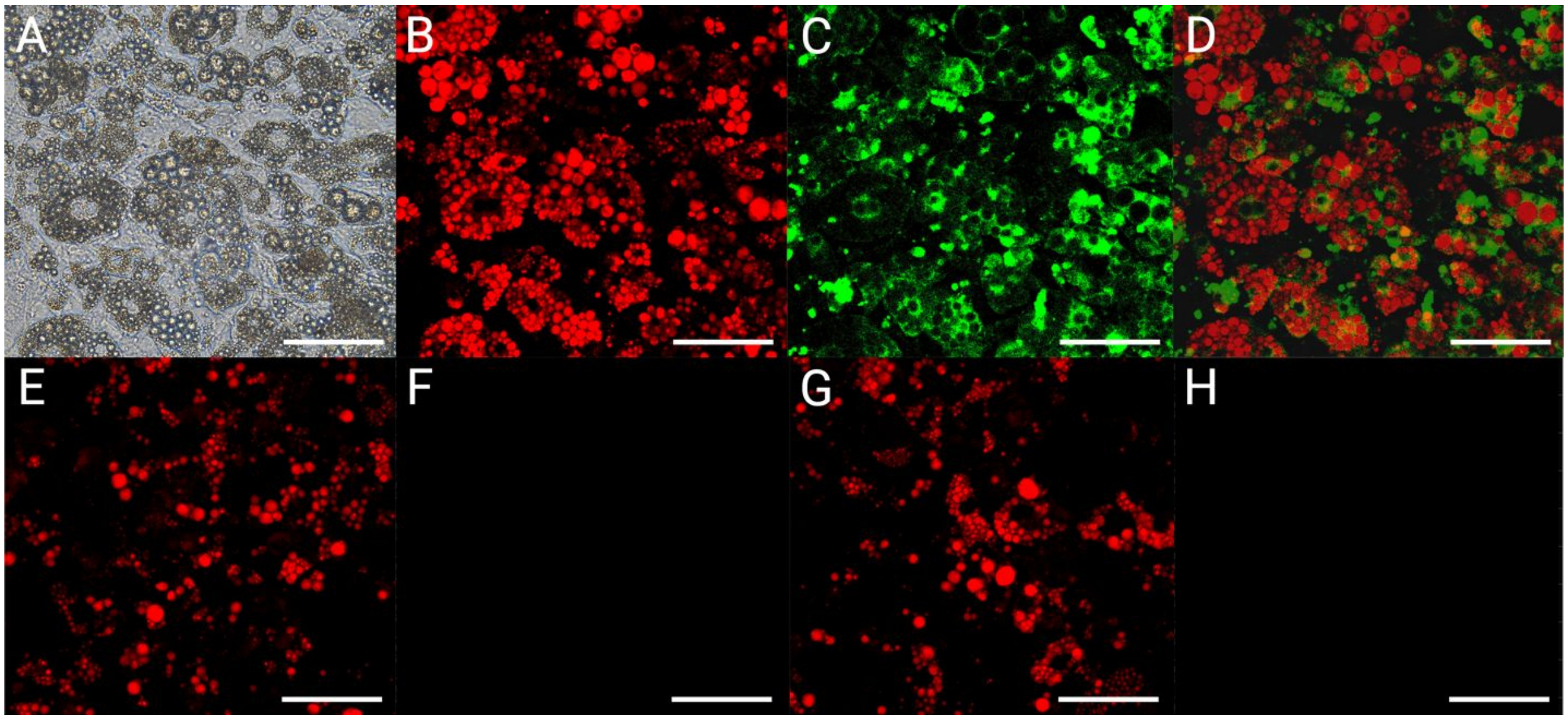

Figure S4. Differentiated 3T3 L1 in vitro *transfection* of FITC-nanoparticles and control conditions. (A-D) Representative images of the same field of differentiated 3T3 L1 cells: (A) Brightfield image of differentiated *3T3 L1* cells with high lipid content, (B) BODIPY lipid (**red**), (C) FITC-nanoparticles (**green**), and (D) fluorescence overlay of intracellular FITC-nanoparticles (**green**) and BODIPY-stained lipids (**red**). (E-F) Untreated control cells from the same field if view: (E) BODIPY lipid (**red**) and (F) FITC fluorescence (**green**), which shows no detectable fluorescence. (G-H) Cells treated with nanoparticles(blank) from the same field of view: (G) BODIPY lipid (**red**) and (H) FITC fluorescence (**green**), which also shows no detectable fluorescence. Scale bar = 50 μm.

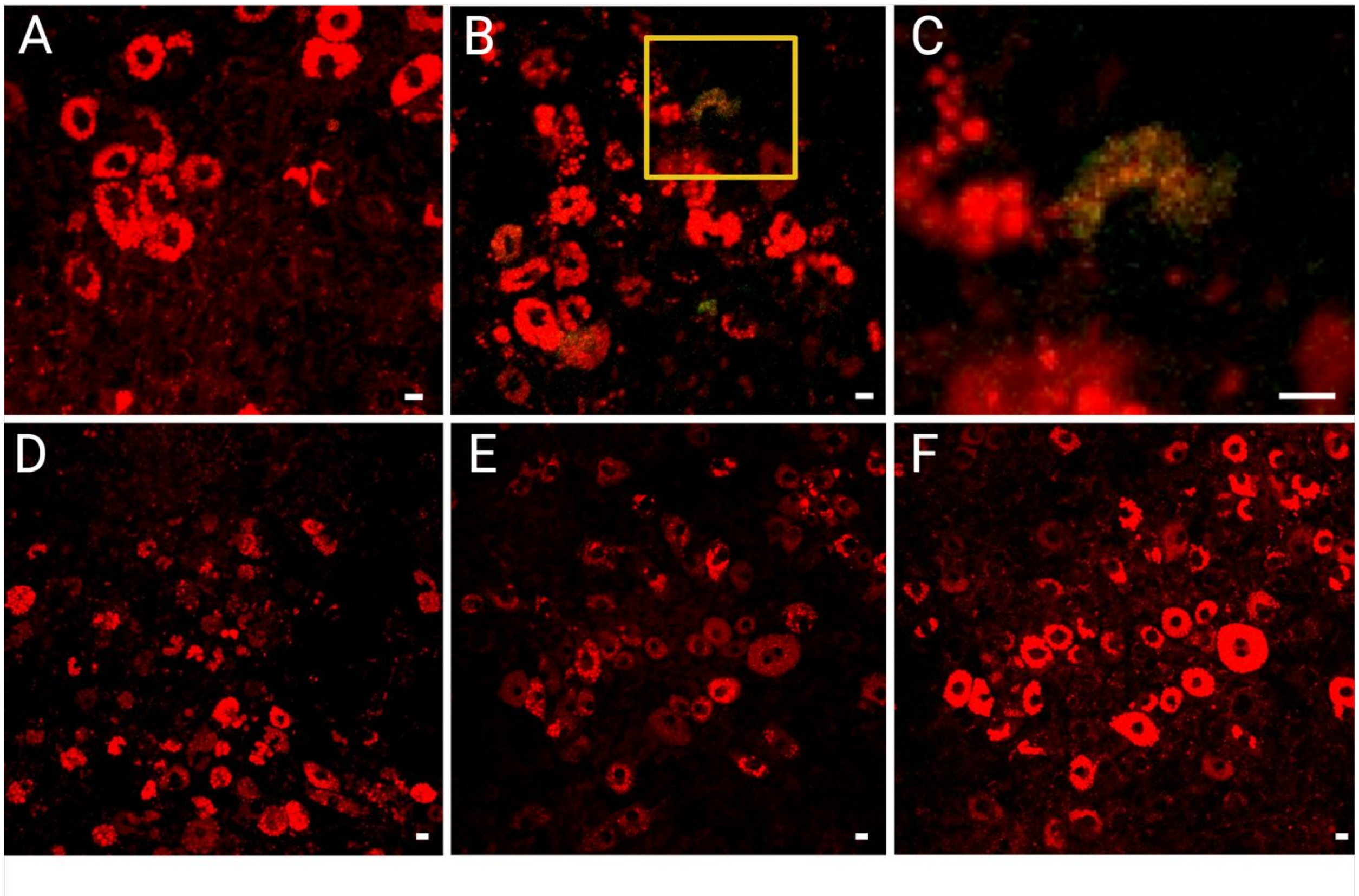

Figure S5. Differentiated 3T3 L1 in vitro *application* of HNE(GFP) and sustained GFP *release* over 8 days. (A-C) Representative images of cells *administered* HNE(GFP): (A) Fluorescence overlay of HNE(GFP) *released* (**green**) and BODIPY-stained lipids (**red**) one day after emulsion *application*, (B) fluorescence overlay of GFP *released* (**green**) and BODIPY-stained lipids (**red**) eight days after emulsion *application*, and (C) magnification of GFP *released* eight days after emulsion *application* (**yellow box**). (D) Untreated control cells (day 8). (E-F) Cells with HNE(blank) *application* at day 1 (E) and day 8 (F). Control conditions show only BODIPY lipid signal (**red**). Scale-bar = 10 µm.
